# Integrated multi-omics reveals dysbiosis in hemodialysis patients: A multi-center study

**DOI:** 10.1371/journal.pone.0355698

**Published:** 2026-08-13

**Authors:** Xinyue Zhang, Dan Yu, Yupeng Cui, Yanqing Chi, Zhenyu Yan, Yang Song, Liping Hou, Jin Qin, Jingjing Zhang, Yuanyuan Wang, Wei Wei, Hailing Di

**Affiliations:** 1 School of Public Health, Hebei Medical University, Shijiazhuang, China; 2 Department of Clinical Nutrition, The Third Hospital of Hebei Medical University, Shijiazhuang, China; 3 Department of Nephrology, The Third Hospital of Hebei Medical University, Shijiazhuang, China; 4 Department of Clinical Nutrition, Xingtai People 's Hospital, Xingtai, China; 5 Department of Clinical Nutrition, Dingzhou People 's Hospital, Dingzhou, China; 6 Department of Clinical Nutrition, Harrison International Peace Hospital, Hengshui, China; 7 The Biobank, Third Hospital of Hebei Medical University, Shijiazhuang, China; 8 Clinical Biochemistry Lab, Third Hospital of Hebei Medical University, Shijiazhuang, China; Zhejiang University, CHINA

## Abstract

**Introduction:**

The gut microbiome-metabolome interplay in hemodialysis (HD) patients remains poorly characterized. Using multi-omics approaches, we compared HD patients with healthy controls (HC) to identify microbial signatures, metabolic perturbations, and their integrated correlations.

**Methods:**

This case-control study included 192 participants (96 HD-HC pairs under identical dietary and living conditions). The gut microbiota composition was analyzed using 16S ribosomal RNA gene sequencing, and fecal metabolomes were analyzed using ultra-high-performance liquid chromatography and high-resolution mass spectrometry (UPLC-HRMS). A multi-omics analysis was conducted utilizing Spearman correlation analysis, Mantel test analysis, and differential functional pathway analysis.

**Results:**

We observed significant differences in gut microbiota composition between the HD and HC groups, such as *Ruminococcus* and *Bifidobacterium*. Comparative analysis revealed 497 significantly altered metabolites in the HD group versus HC, primarily associated with amino acid, vitamin, lipid, purine, and pyrimidine metabolisms. ROC analysis identified 4-pyridoxic acid, nudifloramide, imidazoleacetic acid, ascorbic acid, and tocopheronic acid as potential diagnostic biomarkers (AUC > 0.8, *p* < 0.01). Integrated multi-omics analysis revealed correlations between *Ruminococcus* and metabolites such as Docosapentoic acid (DPA), 13 − EPAHAAB (EPA), and tryptamine, with shared differential pathways in bile secretion, caffeine metabolism, gastric acid secretion, and vitamin B6 metabolism.

**Conclusion:**

Hemodialysis patients exhibited significant alterations in gut microbiota composition and metabolic profiles (amino acid, vitamin, and lipid metabolism) compared with healthy controls, with demonstrated microbiome-metabolome interactions and shared functional pathways. The potential diagnostic and therapeutic value of these differential features warrants further exploration and external validation.

## Introduction

Chronic kidney disease (CKD) has emerged as a growing public health concern [1]. Globally, hemodialysis (HD) is the predominant modality, used by approximately 85% of dialysis patients, whereas peritoneal dialysis (PD) accounts for about 11%, with substantial variation across countries [2,3]. According to the latest data from the China National Renal Data System (CNRDS), HD constituted approximately 86.8% of the dialysis population in China, and the number of patients receiving maintenance hemodialysis reached 1,027,267 by the end of 2024 [4]. Moreover, the prevalence of malnutrition in HD patients is as high as 30.0% ~ 66.7% [5]. Therefore, it is crucial to find targets to improve nutritional and disease status in HD patients.

The microbial ecology of the human gastrointestinal tract (GIT) is highly diverse. However, uremia causes gut dysbiosis-a disorder that alters the normal makeup and activity of the gut microbiota. Taxonomic and functional changes in the GIT microbiome have been linked to numerous illnesses, including inflammatory bowel disease (IBD) [6,7], malnutrition [8,9], cardiovascular events [10], higher mortality risk [11-12], and metabolic disorders [13]. Increased α-diversity, a measure of bacterial evenness and richness, is generally linked to better health outcomes [14]. Recent studies have reported that patients receiving hemodialysis exhibit alterations in gut microbial diversity; however, the direction of change remains inconsistent. Some studies have observed a dramatic decrease in gut microbial diversity in HD patients, which is strongly associated with malnutrition and inflammation [15,16], whereas others have reported increased richness or no significant differences in diversity indices [17,18]. These discrepancies may arise from differences in study populations, dietary habits, living environments, and insufficient control of confounding factors. Even after controlling for influencing variables including age, BMI, dietary habits, and living environment, it remains unclear whether consistent differences in gut microbiota exist between HD patients and healthy controls (HC).

Metabolomics systematically captures dynamic changes in small-molecule metabolites. In the context of disease, it can uncover disrupted metabolic pathways and support the identification of therapeutic targets and early diagnostic biomarkers [19–21]. Although metabolomics has been used to identify indicators linked to the advancement of chronic kidney disease, most studies have focused on serum metabolomics [22,23]. Morgan E. Grams and colleagues discovered that several metabolites, including kynurenate, hippurate, and homocitrulline, are associated with the advancement of chronic kidney disease [24]. Shengyuan Luo et al. identified 58 serum metabolites with cross-sectional relationships to proteinuria; several of these metabolites were also connected to the advancement of chronic kidney disease (CKD) [25]. While metabolomics has emerged as a promising area of research in human diseases, our understanding of fecal metabolic activity in patients receiving hemodialysis remains limited, and the differences in metabolism and metabolic pathways between HD patients and healthy individuals are poorly characterized. Furthermore, in the context of CKD, coordinated multi-omics research is crucial to improving our comprehension of the complex interactions between host health and microbial diversity.

Therefore, the primary objective of this study was to evaluate differences between HD patients and HC individuals in gut microbial diversity, fecal metabolite profiles, and associated metabolic pathways using a 1:1 matched case-control design. The secondary objective was to perform integrated multi-omics analysis to examine correlations among identified microbial species, metabolites, and functional pathways, thereby generating hypotheses for future mechanistic and translational studies.

## Materials and methods

### Study design and patient population

This study was a multicenter prospective 1:1 matched case-control study. Hemodialysis (HD) patients were recruited from four dialysis centers in Hebei Province, China, including one provincial and three municipal hospitals. Participants were recruited through two approaches: (1) public posters in dialysis centers, and (2) face-to-face interviews by trained research staff. Data were prospectively collected between July 2023 and April 2024. This study was approved by the Ethics Committee of the Third Hospital of Hebei Medical University (Approval No. W2024-012-1). All participants provided written informed consent. All methods were performed in accordance with the relevant guidelines and regulations. No waiver of informed consent was granted, as all participants were competent and willing to sign the consent form.

**Matching strategy.** Each HD patient was paired with a healthy control living in the same household and sharing the same diet and living environment. This household paired design is grounded in the principle that cohabitation substantially reduces individual variability in gut microbiota composition and enhances statistical power [26]. Similar designs have been successfully used in previous microbiome studies [27–28]. Age, sex, height, weight, and BMI were not used as matching criteria; they are reported in S1 Table only as descriptive characteristics of the two groups after matching.

**Inclusion criteria for HD patients.** (1) age 18–80 years; (2) regular hemodialysis for > 3 months; (3) adequate dialysis adequacy (Kt/v > 1.2); **Inclusion criteria for HC**: (1) living and eating with the corresponding HD patient and providing written informed consent; (2) age within ± 2 years of the patient; (3) willingness to provide a fecal sample and comply with the study procedures.

**Exclusion criteria for both groups.** (1) severe cardiopulmonary, hepatic, or renal diseases, malignancies, or gastrointestinal disorders; (2) autoimmune diseases or long-term immunosuppressant use; (3) peritonitis or other severe infections within the past month; (4) surgery within the past month; (5) fever, abdominal pain, or diarrhea within the past two weeks; (6) use of probiotics or prebiotic supplements; (7) use of antibiotics within 3 months prior to enrollment; **Additional exclusion criteria for HC**: (1) use of antibiotics or probiotics within 3 months prior to enrollment; (2) history of travel to a region geographically distinct from the dialysis patient cohort; (3) gastrointestinal disorders (e.g., diarrhea) preventing stool collection.

### Dietary intake survey and evaluation

We conducted a 3-day, 24-hour dietary recall survey for participants through face-to-face interviews [29]. Average daily energy and nutrient intake were determined by using specialized nutrition software (Nutrition Clinic Management System, V1.27.2-N, Shanghai Weihu Computer Tec Co., Ltd). The case and control groups cohabited and shared identical dietary sources, with no significant differences in mean daily per capita nutrient intake (all *p* > 0.05, S2 Table), confirming that the household matching strategy successfully achieved comparable dietary intake between groups and effectively minimized diet as a confounding factor.

### Clinical data and laboratory measurements

Anthropometric measurements and body composition evaluations were performed by trained professionals subsequent to the mid-week hemodialysis treatment. An automatic height and weight meter (InBody BSM370, Biospace, Korea) was used to measure our height and weight. Body mass index (BMI) was calculated as weight divided by the square of height in meters: BMI = weight (kg) / [height (m)]². During the on-site study, clinical data were gathered. Every participant had to take measurements while wearing light clothing and after a 12-hour fast. Following an 8-hour fast, blood was taken right before the midweek dialysis session. An automated biochemistry analyzer (BS-360S, Catalog No. 116-022-100; Shenzhen Mindray Bio-Medical Electronics Co., Ltd., Shenzhen, China) was used to perform blood biochemistry studies, such as albumin (ALB), prealbumin (PA), uric acid (UA), etc. MIS (Malnutrition-inflammation score) [30–31] and Neutrophil-lymphocyte Ratio (NLR) were used for the evaluation of the nutritional status and the inflammation status.

### Fecal sample collection

Fecal samples were obtained at home using a disposable stool collection cup and transported to the hospital laboratory and stored in a −80℃ refrigerator immediately. Total fecal microbial DNA was extracted using the Fecal Genome DNA Extraction Kit (Catalog No. AU46111−96; BioTeke Corporation, Beijing, China). DNA quantification was performed utilizing Qubit (Invitrogen, USA).

### 16S rRNA gene amplification and sequencing

The V3-V4 hypervariable regions of the bacterial 16S rRNA gene were amplified using bar-coded primers 341F (5’-CCTACGGGNGGCWGCAG-3’) and 805R (5’-GACTACHVGGGTATCTAATCC-3’) [32]. PCR reactions were performed in a total volume of 25 μL containing 12.5 μL of 2 × Phusion High-Fidelity PCR Master Mix (Catalog No. F531L; Thermo Fisher Scientific, Waltham, MA, USA), 0.2 μM each primer, and 10 ng of template DNA. Thermal cycling conditions were as follows: initial denaturation at 98°C for 30 s; 25 cycles of denaturation at 98°C for 10 s, annealing at 54°C for 30 s, and extension at 72°C for 30 s; followed by a final extension at 72°C for 5 min. PCR amplicons were purified using AMPure XT magnetic beads (Catalog No. A63882; Beckman Coulter Genomics, Danvers, MA, USA). Purified amplicons were pooled in equimolar concentrations and paired-end sequenced (2 × 250 bp) on an Illumina NovaSeq 6000 platform (Illumina, Inc., San Diego, CA, USA) by LC-Bio Technology Co., Ltd. (Hangzhou, China).

### Bioinformatics analysis of 16S rRNA data

Raw sequencing data were demultiplexed, and sequencing primers were removed using Cutadapt (version 1.9). Paired-end reads were merged using FLASH (version 1.2.8) with default parameters (minimum overlap = 10 bp, maximum mismatch density = 0.25). Quality filtering was performed using fqtrim (version 0.94) with a sliding-window algorithm (window size = 4, average quality threshold = Q20). Reads shorter than 100 bp or containing more than 5% ambiguous bases (“N”) were discarded. Chimeric sequences were identified and removed using VSEARCH (version 2.3.4) with the “uchime_denovo” algorithm. Amplicon sequence variants (ASVs) were inferred using the DADA2 pipeline (version 1.18.0) within the QIIME2 framework (version 2021.11). Taxonomic assignment of ASVs was performed using the QIIME2 feature-classifier plugin (classify-sklearn naive Bayes taxonomy classifier) against two reference databases: (1) the NT-16S database (Release 20230718; National Center for Biotechnology Information, Bethesda, MD, USA) with thresholds of ≥ 90% identity, ≥ 80% coverage, and E-value ≤ 1e-5; and (2) the SILVA database (Release 138; Max Planck Institute for Marine Microbiology, Bremen, Germany) with a minimum confidence threshold of 0.7. Using QIIME2 and the acquired ASV feature sequences, α and β diversities were computed.

### Diversity and statistical analyses

Alpha diversity (Chao1 and Shannon indices) was calculated using the phyloseq package (version 1.38.0) in R (version 4.3.3). Beta diversity was assessed using Bray-Curtis distance matrices and visualized via principal coordinate analysis (PCoA) using the ade4 package (version 1.7–22). Analysis of similarities (ANOSIM) was performed using distance matrices (Bray-Curtis, Jaccard, weighted and unweighted UniFrac) within QIIME2. The differentially abundant genera were identified using the Wilcoxon signed-rank test with Benjamini-Hochberg false discovery rate (FDR) correction. A genus was considered significantly different if FDR-adjusted *p* < 0.05. The distances between samples were ranked to determine the within-group and between-group differences, and permutation tests were used to determine the statistical significance of the initial between-group differences.

### Metabolome sample preparation

Fecal samples (50 mg each) were thawed on ice and extracted with 0.5 mL of cold 80% methanol (HPLC grade, Catalog No. 34860; Honeywell, Muskegon, MI, USA) in ultrapure water (Milli-Q, 18.2 MΩ·cm; Merck, Darmstadt, Germany). The mixture was vortexed for 30 s and incubated at −20°C for 30 min, then centrifuged at 20,000 × g for 15 min at 4°C. The supernatant was transferred to a fresh tube and vacuum-dried. Dried extracts were reconstituted in 100 μL of 50% methanol and stored at −80°C until analysis. A pooled quality control (QC) sample was prepared by mixing 10 μL of each extracted sample and analyzed every 10 injections to monitor system stability.

### LC-MS analysis

Chromatographic separation was performed on an UltiMate 3000 UPLC system (Thermo Fisher Scientific, Bremen, Germany) equipped with an ACQUITY UPLC HSS T3 column (100 mm × 2.1 mm, 1.8 μm; Catalog No. 186003539; Waters, Milford, MA, USA) maintained at 40°C. Mobile phase A consisted of 5 mmol/L ammonium acetate and 5 mmol/L acetic acid in water; mobile phase B was acetonitrile. The flow rate was 0.35 mL/min with the following gradient: 0–0.8 min, 2% B; 0.8–2.8 min, linear increase to 70% B; 2.8–5.0 min, increase to 90% B; 5.0–5.5 min, increase to 100% B; 5.5–5.7 min, hold at 100% B; 5.7–7.6 min, decrease to 2% B; and 7.6–10 min, re-equilibrate at 2% B. Mass spectrometry was performed on an Orbitrap Exploris 120 mass spectrometer (Catalog No. BRE725536; Thermo Fisher Scientific, Bremen, Germany) equipped with a heated electrospray ionization (HESI) source operating in both positive and negative ion modes. Full-scan spectra were acquired at a resolution of 70,000 over an m/z range of 70–1050, with an automatic gain control (AGC) target of 3 × 10⁶ and maximum injection time of 100 ms. Data-dependent acquisition (DDA) selected the top three most intense ions for fragmentation at a resolution of 17,500 (AGC target 1 × 10⁵, maximum injection time 80 ms). A quality control (QC) sample, which was made as a pooled combination of all research samples, was examined after every ten sample injections in order to track the stability and repeatability of the LC-MS system during the analytical run.

### Metabolomics data processing and statistical analysis

Raw LC-MS data were converted to mzXML format and processed using XCMS (version 3.18.0) in R (version 4.3.3) for peak detection, alignment, retention time correction, and annotation of isotopes and adducts. Metabolite annotation was performed by matching accurate m/z values and fragmentation patterns against the HMDB (version 5.0) and KEGG (release 2023) databases using CAMERA (version 1.46.0) and metaX (version 1.6.0) packages. Data were normalized by total ion current. Principal component analysis (PCA) was performed using metaX. Partial least squares discriminant analysis (PLS-DA) was performed using the ropls package (version 1.30.0) with 7-fold cross-validation to prevent overfitting; variable importance in projection (VIP) scores were calculated. Differential metabolites were identified using the following criteria: paired samples t-test (on log2-transformed abundance values) with *p* < 0.05, absolute fold change (FC) ≥ 1.2 (FC ≥ 1.2 for up-regulated metabolites or FC ≤ 0.833 for down-regulated metabolites), and variable importance in projection (VIP) ≥ 1 from PLS-DA. FC was calculated as mean abundance in the HD group divided by mean abundance in the HC group. An FDR was used to adjust the *p*-value for multiple testing (Benjamini–Hochberg). A nominal *p* < 0.05 (paired samples t-test) was used as the initial threshold, and Benjamini-Hochberg FDR-adjusted *p*-values were calculated for multiple testing but not used as a strict inclusion criterion. MetaX was used to do supervised PLS-DA in order to distinguish between the various variables between groups. The VIP value was determined. The selection of significant features was done using a VIP cut-off value of 1.0. Pathway enrichment analysis was performed using the hypergeometric test against KEGG pathways via the clusterprofiler package (version 4.8.0) in R. Receiver operating characteristic (ROC) curves were generated using the pROC package (version 1.15.0) to evaluate diagnostic performance of individual and combined metabolites. We selected the most significant Top 5 of the differential metabolites and drew its separate ROC curve, and calculated the total ROC curve through a logistic regression model.

### Correlation analysis of microbiome and metabolome

Correlation analysis of differential metabolites (FC ≥ 1.2 or FC ≤ 0.833 and *p* < 0.05 and VIP > 1 from metabolomic analysis) and differential species (*p* < 0.05 from microbiome analysis) was performed, including Spearman correlation analysis, Mantel test analysis, and differential functional pathway analysis. The Spearman correlation coefficient is a nonparametric measure of rank correlation. The correlation heatmap were drawn based on results of the Spearman correlation analysis (*p < 0.05, **p < 0.01). The Mantel test was an analysis method for determining the correlation between two sets of distance matrices (rather than two sets of variable matrices).

### Statistical analyses

Frequencies and percentages representing categorical data were compared using the chi-square test. Continuous data with or without a normal distribution were compared using means ± standard deviations or medians and interquartile ranges (IQR). Based on our household paired design, the Paired Samples t-test (for normally distributed data) and the Wilcoxon signed-rank test (for non‑normally distributed data) were employed. Statistical power verification. Post-hoc power analysis was performed using the web-based tool for case-control microbiome studies. With a sample size of 96 matched pairs, a two-sided alpha of 0.05, and moderate effect sizes (Cohen‘s d = 0.4–0.5), our study achieved > 85% power to detect the observed differences in gut microbial diversity and differential metabolites. Statistical significance was defined as two-tailed *p*-values less than 0.05. All statistical analyses were performed using SPSS version 26.0 (IBM Corp., Armonk, NY, USA) and R version 4.3.3 (R Foundation for Statistical Computing, Vienna, Austria). The following R packages were used: phyloseq (v1.38.0), ade4 (v1.7-22), metaX (v1.6.0), ropls (v1.30.0), CAMERA (v1.46.0), pROC (v1.15.0), and clusterProfiler (v4.8.0).

## Results

### Patient characteristics

S1 Table provides a summary of the baseline characteristics of 96 hemodialysis (HD) patients and 96 healthy controls (HC) at the time of sample collection.

### Gut microbial diversity between hemodialysis patients and healthy controls

We analyzed and compared the microbiomes of 192 fecal samples from 96 hemodialysis patients and 96 healthy controls. To assess the sequencing depth, rarefaction curves were produced; when the curves plateaued, it meant that enough sequencing had been done for a trustworthy analysis. Statistically significant variations between the groups were shown by α-Diversity (S9 Fig). The Chao1 index values for HD were significantly higher (*p* < 0.001), but the Shannon index revealed a negligible (*p* = 0.18), diversity-related difference between the two groups. HD patients have higher abundance of gut microbiota than their healthy controls (HC). However, there is no difference in the diversity of gut microbiota. S1 Fig **e-f** illustrated the differences (β-diversity) in gut microbiota composition among samples from distinct groups, as depicted through unconstrained principal coordinate analysis (PCoA). And analysis of similarities showed that the grouping was statistically significant and reliable (*p* = 0.001, R = 0.1625). Select the species classification of top 30 abundance, and the composition differences of dominant species between the two groups at the phylum level were shown with a stacked bar chart (S1 Fig). The most in both groups were *Firmicutes*, and the HD group was higher. *Verrucomicrobiota* also accounted for more in the HD group. However, the amount of *Actinobacteria* and *Bacteroidota* is significantly higher in the HC group.

**Fig 1:**
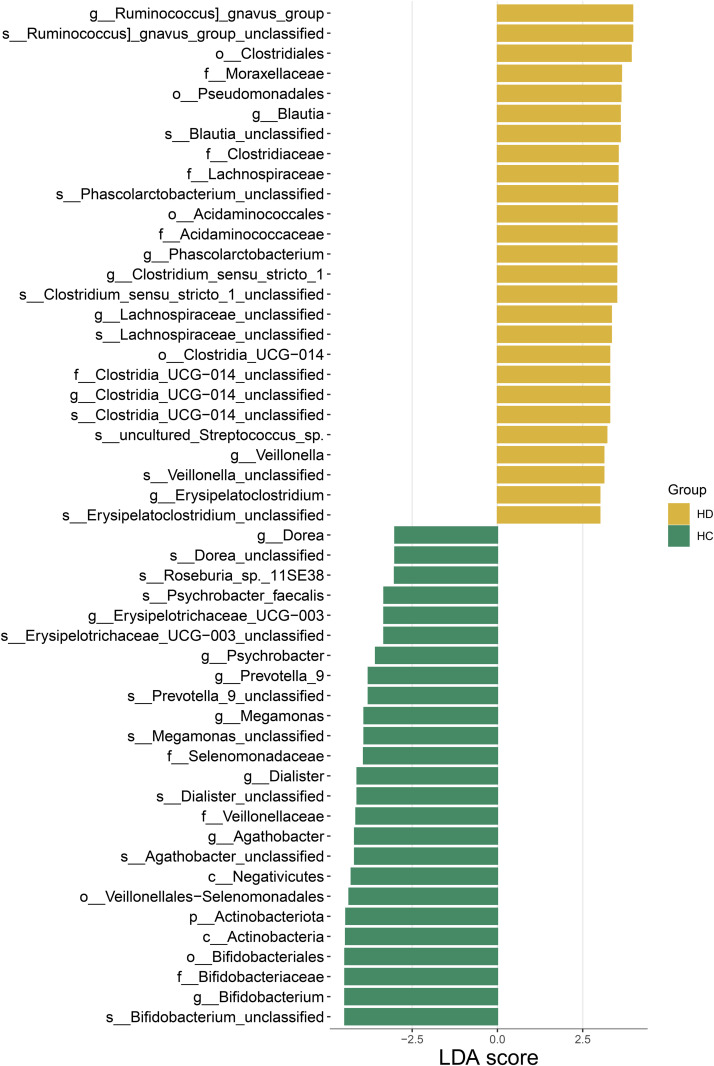
LDA score showing differentially abundant taxa of the gut microbiota. Differential taxon features identified by LEfSe according to hemodialysis patients and healthy controls after grouping (LDA score > 3, *p* < 0.05). Yellow and green bars represent taxon features with significantly higher expression in HD and HC groups, respectively. And the length represents LDA score. LDA linear discriminant analysis, LEfSe linear discriminant analysis effect size.

Sankey plots (S2 Fig) showed the species with abundance top10 (genus) and its affiliated phylum information. Both groups account for the largest proportion of *Bifidobacterium.* And some bacteria were more in the HD group than in HC: *Escherichia-shigella, Akkermansia, Streptococcus, Subdoligranulum,* and *Ruminococcus]_gnavus_group*. 51 distinguishing taxon features across various taxonomic levels between the two groups were revealed by the LEfSe analysis (Fig 1). At the level of genus, *Ruminococcus*, *Blautia*, *Phascolarctobacteriu*, *Clostridium_sensu_ stricto_1*, *Lachnospiraceae. Clostridia_UCG − 014_unclassified*, *Veillonella*, and *Erysipelato -clostridium* were enriched in HD patients. In contrast, HC group demonstrated a higher relative abundance of *Dorea*, *Erysipelotrichaceae_UCG-003*, *Psychrobacter*, *Prevotella_9*, *Megamonas*, *Dialister*, *Agathobacter*, and *Bifidobacterium*. Indicator species analysis found species that may serve as biomarkers in different groups (S3 Fig). Obviously, *Dialister* was considerably enriched in the HC group (*p* < 0.01), whereas *Blautia* and *Fusicatenibacter* were significantly enriched in the HD group. *Ruminococcus_gnavus_group*, *Clostridium_sensu_stricto_1*, *Phascolarctobacterium*, *Streptococcus*, *Roseburia*, and *Subdoligranulum* are also members of the HD group. HC group also enriched with *Megamonas*, *Erysipelotrichaceae_UCG-003*, *Agathobacter*, *Prevotella_9*, and *Bifidobacterium.* This is consistent with the statistical results of LEfSe analysis.

### Overall metabolomics analysis of fecal samples

**Fig 2:**
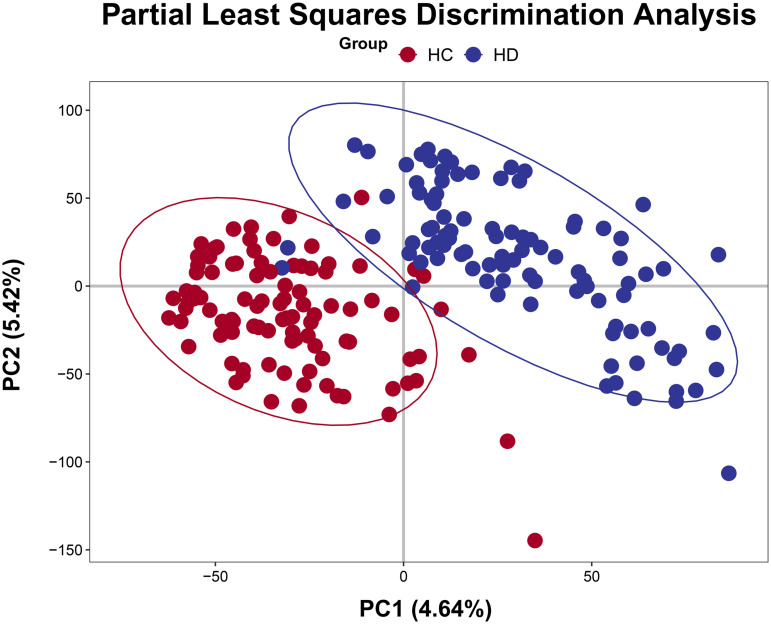
Partial least squares discriminant analysis (PLS-DA) of fecal metabolomics data from hemodialysis patients and healthy controls. The blue dots represented HD patients and the red dots represented healthy controls in the two-dimensional PLS-DA score plots (a). R^2^ regression line is red, Q^2^ regression line is blue (b). If the R² regression line lies above the Q² line and the y-intercept of the Q^2^ regression line is less than 0, the model can be considered free from overfitting.

**Fig 3:**
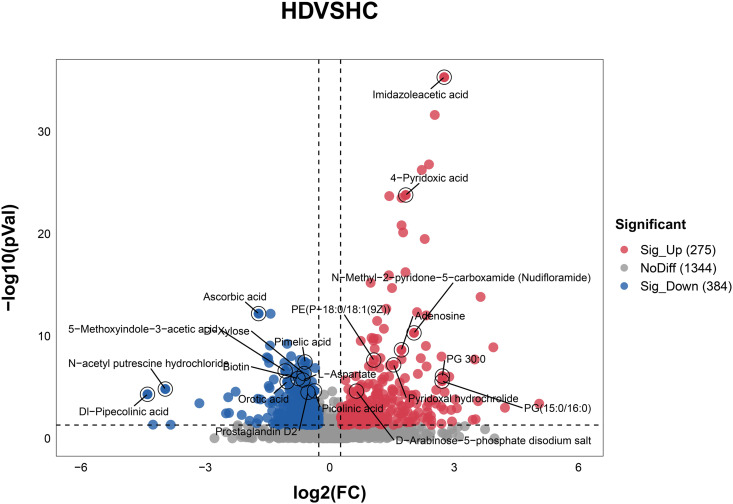
Overall distribution of differential metabolites in hemodialysis patients and healthy controls. Volcano plots were drawn for all metabolites in differential expression analysis. Red represented upregulated significantly differentially expressed metabolites, blue represented downregulated, and gray dots represented non-significantly differentially expressed metabolites.

192 fecal samples from the HD patients (96) and HC group (96) had their metabolomes analyzed and contrasted. Using R (version 4.3.3), a total of 2275 molecular characteristics were gathered and statistically examined. With an intercept of Q2 = −0.32 and an R2 = 0.67, PLS-DA showed that the HD and HC samples had different distributions, demonstrating the model's dependability and lack of overlifting (Fig. 2). Differential metabolites were filtered based on FC ≥ 1.2 or FC ≤ 0.833, *p* < 0.05, and VIP ≥ 1, which were then displayed using a heatmap (S4 Fig). When comparing the feces of the HD group to those of the HC group, 497 important metabolites that were involved in the metabolism of amino acids, vitamins, lipids, purines, and pyrimidines were changed. Here we show only the top 20 most significant metabolites (S3 Table). The levels of Imidazoleacetic acid, 4-Pyridoxic acid, N-Methyl-2-pyridone-5-carboxamid, PE(P-18:0/18:1(9Z)), Adenosine, Pyridoxal hydrochrolide, D-Arabinose-5-phosphate disodium salt, PG 30:0, and PG(15:0/16:0) were increased in fecal samples of HD. Whereas, the levels of Ascorbic acid, Prostaglandin D2, Dl-Pipecolinic acid, Biotin, D-Xylose, 5-Methoxyindole-3-acetic acid, Pimelic acid, L-Aspartate, Picolinic acid, Orotic acid, and N-acetylputrescine hydrochloride were decreased in HD (Fig 3).

### Metabolic pathway analysis

To identify biologically significant patterns within fecal metabolomics data, pathway analysis was conducted utilizing the KEGG metabolic database in conjunction with the Hypergeometric test (*p* < 0.05). The top20 perturbed metabolic pathways ranked with *p*-values are shown in a bubble plot (S5 Fig). The rich factor results showed that the Glycosylphosphatidylinositol (GPI)-anchor biosynthesis pathway had the greatest enrichment degree. The second is Cholesterol metabolism pathway. The pathway of Autophagy, Glycerophospholipid metabolism, Bile acid and Bile secretion, Sphingolipid signaling pathway, digestion and absorption of protein and vitamin, and Tryptophan metabolism also differed in the fecal samples between the two groups.

### Metabolite diagnostic performance

**Fig 4:**
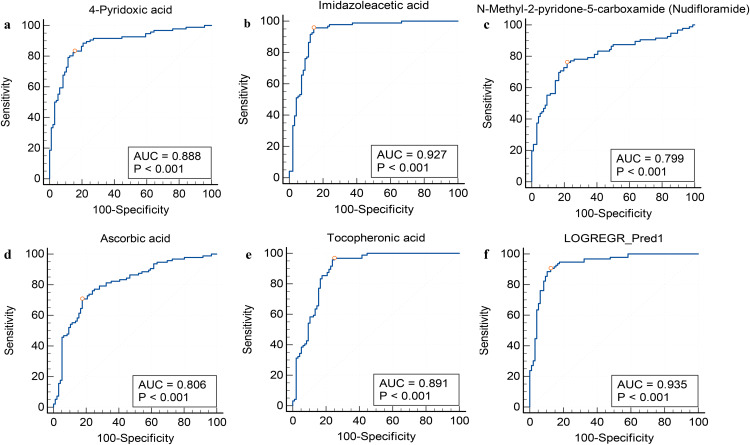
ROC analysis of potential biomarkers for differentiating hemodialysis patients from healthy controls. 4-Pyridoxic acid showed an AUC of 0.888 (95% CI: 0.834-0.929, *p* < 0.001) (a); the N-Methyl-2-pyridone-5-carboxamide presented an AUC of 0.799 (95% CI: 0.735-0.853, *p* < 0.001) (b); Imidazoleacetic acid presented an AUC of 0.927 (95% CI: 0.881-0.960, *p* < 0.001) (c); Ascorbic acid presented an AUC of 0.806 (95% CI: 0.742-0.859, *p* < 0.001) (d); Tocopheronic acid presented an AUC of 0.891 (95% CI: 0.838-0.931, *p* < 0.001) (e); the combined model performed an AUC of 0.935 (95% CI: 0.890-0.965, *p* < 0.001) (f). *The data were not normally distributed, therefore the paired Wilcoxon signed-rank test was employed.

After proving that metabolomics is essential for differentiating HD patients from HC controls, we looked into possible HD diagnostic biomarkers. For a preliminary evaluation of their diagnostic performance, the top five metabolites with the highest significant *p*-values were chosen for conventional univariate receiver operating characteristic (ROC) curve analysis. 4-Pyridoxic acid, N-Methyl-2-pyridone-5-carboxamide (Nudifloramide), imidazoleacetic acid, ascorbic acid, and tocopheronic acid had respective area under the curve (AUC) values of 0.888, 0.799, 0.927, 0.806, and 0.891. Fig 4 displayed the metabolites’ and the combined model's corresponding ROC curves. 4-The sensitivity and specificity of pyridoxic acid were 83.33% and 84.37%, respectively, with an AUC of 0.888 (95% CI: 0.834–0.929) (Fig 4a). N-Methyl-2-pyridone-5-carboxamide had a sensitivity of 76.04%, a specificity of 78.12%, and an AUC of 0.799 (95% CI: 0.735–0.853) (Fig 4b). According to Fig 4c, the imidazoleacetic acid had an AUC of 0.927 (95% CI: 0.881–0.960), a sensitivity of 95.83%, and a specificity of 85.42%. According to Fig 4d, the ascorbic acid had an AUC of 0.806 (95% CI: 0.742–0.859), a sensitivity of 70.83%, and a specificity of 82.29%. According to Fig 4e, the tocopheronic acid had an AUC of 0.891 (95% CI: 0.838–0.931), a sensitivity of 96.87%, and a specificity of 75%. The illness results in simultaneous changes to several metabolites. To more accurately forecast the biomarker's value, we compute the overall AUC value of these target metabolites using the logistic regression model. The combined model yielded a *p* < 0.001, a sensitivity of 90.62%, a specificity of 87.5%, and an AUC of 0.935 (95% CI: 0.890–0.965) (Fig 4f).

### Combined analysis of metabolomics and gut microbiomes

We used Spearman correlation analysis on the differential genus and differential metabolites of the HD group vs HC group from microbiome and metabolomics study to further investigate the connection between gut microbial flora and host metabolic phenotype. Correlation heatmap between differential genus and differential metabolites showed that the most relevant ones were *Ruminococcus* and 5,8,11-Etycotriolic acid (ETI), and *Ruminococcus* and Docosapentoic acid (DPA). Secondly, related to *Ruminococcus* is Adrenic Acid, 13-EPAHAAB (EPA), and Tryptamine (S6 Fig). The correlation network among differential genera and metabolites is shown in S7 Fig. We also use the Mantel test to determine the correlation between the two sets of distance matrices (S8 Fig). The network diagram indicated that the correlation between *Glucerabacte* and Naphthalenecarboxae is the most significant, and it is negatively correlated (r < −0.5). Among the other metabolites, the metabolites with the most correlation with the gut microbiota species were 1-Palmitoyl-2-linoleoyl PE and 1-Methyladenine. Comparing the functional pathways with statistical differences in microbiomics and metabolomics, it was found that the differential functional pathways shared by the two omics were as follows: Bile secretion, Caffeine metabolism, Gastric acid secretion, and Vitamin B6 metabolism.

## Discussion

Chronic kidney disease, a growing public health concern, substantially reduces the nutritional status of patients [33]. We carried out a 1:1 paired design under the same diet and living conditions and ruled out age and gender distractions. We revealed the differential microbiota, differential metabolites, and differential functional pathways between hemodialysis (HD) and healthy controls (HC), and we explored potential connections between the two omics.

Changes in the species and distribution of intestinal flora can be defined as gut dysbiosis [34]. We observed that gut microbial richness was significantly different between hemodialysis patients (HD) and healthy controls (HC). On average, HD patients demonstrated higher microbial richness and diversity than HC group. In particular, we discovered that the relative abundance of two beneficial bacteria for humans, *Actinobacteria* and *Bacteroidota* [35–36], was significantly lower in HD than in HC. This suggests that certain taxonomic configurations of the human gut microbiota may represent health-related alterations associated with hemodialysis. Numerous intestinal and extraintestinal conditions, including CKD, obesity, hypertension, and inflammatory bowel disease, have been linked to decreased gut microbial diversity [37–41]. Notably, new metabolomics studies have shown that specific gut-derived uremic toxins are highly and positively linked with gut microbial diversity, despite the fact that a more diverse microbial community is frequently thought to reflect a healthy host-microbiome connection [42–43]. Thus, our results have higher richness in HD, which may be due to the increase in certain toxins and pathogenic bacteria. For example, excessive proliferation of *Firmicutes* may lead to metabolic imbalance; *Clostridium_sensu_stricto* and *Erysipelato-clostridium* as pathogenic bacteria can increase the infection risk in HD patients [44–46]. *Blautia* can maintain the integrity of the intestinal mucosa and regulate intestinal immune function [47–48]. *Blautia* and *Fusicatenibacter* can serve as biomarkers to distinguish HD patients from healthy individuals. *Bifidobacterium* and *Prevotella* were beneficial bacteria [49–50], especially in the HC group, our results corroborate previous findings. Hence, in the context of HD, extensive and comprehensive omics research is needed to deepen our comprehension of the intricate relationships between microbial diversity and host health.

It was feasible to differentiate HD patients from healthy controls in this study because to the chemical profiles of the fecal samples. Following screening, it was discovered that the HD and HC groups had significantly different levels of 497 fecal metabolites. Compared to healthy controls, HD patients had disturbed metabolisms of amino acids, vitamins, lipids, purines, and pyrimidines in connection to dialysis. Certain amino acid levels were changed in HD patients, and the elevated imidazoleacetic acid is a byproduct of histidine metabolism; And those that have been decreased are: Dl-pipecolinic acid, L-Aspartate, and Picolinic acid, they are involved in lysine metabolism, tricarboxylic acid cycle (TCA), and tryptophan metabolism, respectively. This is consistent with Lanzon B's conclusion [51]. In an animal experiment on intestinal flora and metabolites in the development of chronic kidney disease, Liu et al. [52] found that metabolites that were differentially abundant in feces were mainly linked to tyrosine metabolism, histidine metabolism, and the biosynthesis of tryptophan and phenylalanine. Additionally, Thalacker-Mercer's review [53] demonstrated that pathologic circumstances [such as chronic kidney disease (CKD) and chronic obstructive pulmonary disease (COPD)] result in a decrease in circulating amounts of histidine. Pyridoxal hydrochloride and 4-Pyridoxic acid participate in the metabolic process of vitamin B6, and N-Methyl-2-pyridone-5- carboxamide is a metabolite of vitamin B3. Both of these are higher in the HD group. However, Ascorbic acid (Vitamin C) and Biotin (Vitamin B7) were lower than HC group. A deficit of vitamin B6, a rate-limiting coenzyme that is crucial for the production of heme, is frequently observed in patients with chronic renal disease, especially those who need dialysis and after receiving erythropoietin-stimulating agent (ESA) [54]. Kumakura et al. [55] discovered that nicotinamide (B3) can foster the production of adenosine triphosphate (ATP), and reduce kidney inflammation and fibrosis. Dialysis patients frequently have vitamin C shortage, which can be caused by increased vitamin C catabolism in vivo from inflammation, limited consumption of foods high in vitamin C, and dialytic vitamin C clearance [56]. Biotin, a water-soluble vitamin, is crucial as one of the coenzymes in energy production, according to Fujiwara M's study [57]. HD-related cramps may be caused by a biotin deficiency, and biotin's coenzyme actions are hampered by accumulating biotin metabolites. Vitamin B6 and B3 metabolism were active in HD patients. Conversely, vitamin C and biotin metabolism are lower. Moreover, PG(16:0/18:1(9Z)), PG 30:0, and PG(15:0/16:0), as isomers of phosphatidylglycerol (PG), were increased to maintain cell membrane stability and to participate in signaling [13,58]. Adenosine can participate in ATP synthesis and inhibit inflammatory responses, which also increase in HD. The primary potential biomarkers of HD (4-Pyridoxic acid, Nudifloramide, Imidazoleacetic acid, Ascorbic acid, and Tocopheronic acid) found by ROC analysis also confirmed the above important differential metabolites.

We observed metabolic dysregulation with certain metabolic pathways in fecal samples of HD patients, mainly Glycosylphosphatidylinositol (GPI)-anchor biosynthesis pathway, and the pathway of Autophagy, Cholesterol metabolism, Glycerophospholipid metabolism, Bile acid and Bile secretion, Sphingolipid signaling, digestion and absorption of protein and vitamin, and Tryptophan metabolism. GPI-anchored proteins play an important role in cell recognition, signal transduction and immune response, involving a series of enzymatic reactions [59]. Cholesterol is converted into bile acids in the liver through hydroxylation, oxidation and binding reactions, which together regulate the balance of cholesterol metabolism. The metabolism of tryptophan includes the production of kynurenine and other metabolites, the results suggested that the metabolism of the kynurenine pathway may be a significant factor in hemodialysis and a possible target for treatment in HD patients [60–61]. Additionally, the digestion and absorption of proteins and vitamins in the intestine, intestinal autophagy pathway, and the metabolism of glycerophospholipids were all abnormal in HD group. Hence, these highlighted metabolites play an important role in physiological processes such as amino acid metabolism, energy metabolism, nucleic acid synthesis, immune regulation and antioxidant defense. Their metabolic pathways and physiological functions reflect the complex metabolic networks and regulatory mechanisms in the human body.

Based on the correlation analysis of microbiota and metabolomics, of important note, the most relevant combination were *Ruminococcus* and Etycotriolic acid (ETI), *Ruminococcus* and Docosapentoic acid (DPA), and *Ruminococcus* and 13-Epahaab (EPA). *Ruminococcus* is a member of the Firmicutes family, which ferments dietary fiber and generates metabolites such short-chain fatty acids (SCFA). According to Lin TY's study [62], the dialysis group with decreased mortality had increased expression of two SCFA-producing bacteria, Succinivibrio and Anaerostipes. SCFAs have been shown to have a variety of effects on host physiology, such as anti-inflammatory qualities and the preservation of gut integrity [63]. Omega-3 fatty acids, such as DPA and EPA, are known to have anti-inflammatory and hypotriglyceridemic effects, but their dietary equivalents have been shown to have pro-inflammatory qualities [64]. ETI is also a regulator of lipid metabolism. *Ruminococcus* has a clear metabolic association with 3 differential metabolites, which may affect health through regulating lipid metabolism and immunomodulation and anti-inflammatory synergistic effects [65]. The differential functional pathways of HD vs HC were identified from microbiome and metabolomics, respectively. However, the following pathways are mutual to both groups: Bile secretion, Caffeine metabolism, Gastric acid secretion, and Vitamin B6 metabolism. This is consistent with our KEGG pathway enrichment conclusion. Through reasonable thinking, HD patients are often accompanied by abnormal liver function, which indirectly affects bile secretion, and dialysis will cause slowed caffeine metabolism and reduce the efficiency of metabolic waste removal. Dialysis dietary restrictions may lead to insufficient vitamin B6 intake, and uremia toxins may interfere with the activation and utilization of vitamin B6. Similarly, uremia toxins may damage the gastric mucosal barrier, leading to abnormal gastric acid secretion [66–67].

### Innovations

First, the 1:1 household-paired design is a key innovation. Unlike most previous studies, which did not control for diet or living conditions (major confounders of gut microbiota), we matched each HD patient with a cohabiting HC under the same diet. This minimizes confounding and allows us to attribute observed differences to HD itself rather than lifestyle factors. Second, we performed an integrated analysis of the fecal microbiome and metabolome, a more comprehensive approach than previous studies that relied on 16S sequencing or serum metabolomics alone. This allowed us to identify shared functional pathways across both omics layers, a cross‑omics convergence rarely reported in HD patients. Third, the fecal biomarkers we identified achieved high diagnostic accuracy (AUC up to 0.927). They are non‑invasive and directly reflect gut microbial metabolism, offering practical advantages over serum‑based markers for screening and monitoring, though external validation is still needed.

### Clinical translation feasibility

Our biomarkers are fecal, not serum. This makes collection non‑invasive and easy for patients to do at home, which supports repeated testing. Fecal markers also directly reflect gut microbial activity, whereas serum markers capture mixed host‑microbial metabolism after absorption. However, fecal markers come with challenges. Stool consistency, transit time, and sampling can cause variability. The current LC‑MS method is not routine in most labs and is relatively costly. A more practical next step would be to develop targeted assays, such as ELISA or point-of-care kits. This still requires external validation.

Our results suggest that vitamin B6, B3, C, and biotin supplementation might help restore metabolic balance, while increasing dietary fiber could support *Bifidobacterium* and short‑chain fatty acid(SCFA) production. We could also consider using possible probiotics, such as *Bifidobacterium*. These strategies aim to counter the observed dysbiosis and metabolic shifts, but need validation specifically in HD patients.

Our study has several strengths. To the best of our knowledge, this is the first published report to select a healthy control group according to the same diet and living conditions for a 1: 1 matched case- control study in the hemodialysis population. We have advanced amplicon sequencing and state-of-the-art metabolomics platform, and we perform rigorous statistical analysis based on a sufficient sample size. In addition to analyzing the microbiome and metabolome separately, we also analyzed multi-omic correlations. There are various limitations to our investigation. First, due to the observational design, our findings cannot indicate causal links. Second, only baseline fecal samples were taken. However, since microbial diversity may decrease with time in HD patients, a higher baseline microbial diversity could bias the study results in favor of the null hypothesis. Third, the long‑term effects of past antibiotic use on gut microbiota cannot be fully excluded. Fourth, limited by the depth and scope of the study, we have not yet conducted in-depth research on the underlying mechanisms that affect the disease process and prognosis of patients. We also acknowledge several technical limitations. Using 16S rRNA sequencing instead of shotgun metagenomics limited species level resolution and functional prediction. The absence of serum metabolomics means we cannot directly connect fecal changes to the host's systemic metabolic status. Since all participants came from Hebei Province, our findings may not generalize to other regions. In the future research process, it is imperative to further delve into the characteristic profiles of the gut microbiome and gut metabolome in HD patients, explore their potential value as diagnostic markers, and use this as an entry point to explore possible therapeutic intervention targets. This is a highly prospective and important future direction.

## Conclusion

In this study, we employed a household-paired design (HD patients and cohabiting healthy controls sharing the same diet and living environment) combined with 16S rRNA sequencing and fecal metabolomics. The main conclusions are as follows: First, HD patients exhibited higher gut microbial richness (Chao1 index) but no significant difference in Shannon diversity compared with controls, with enrichment of *Ruminococcus* and depletion of *Bifidobacterium*. Second, A total of 497 fecal metabolites were significantly altered in HD patients, predominantly involving amino acid, vitamin, and lipid metabolism. Third, Integrated multi-omics analysis revealed correlations between specific gut bacteria and metabolites, and identified four shared dysregulated functional pathways: Bile secretion, Caffeine metabolism, Gastric acid secretion, and Vitamin B6 metabolism. Fourth, The fecal metabolites 4-pyridoxic acid, imidazoleacetic acid, and ascorbic acid showed high diagnostic accuracy and may serve as non‑invasive candidate biomarkers for HD.

### Practical application

It is meaningful to delve into the characteristic profiles of the gut microbiome and gut metabolome in HD patients, explore their potential value as diagnostic markers, and use this as an entry point to explore possible therapeutic intervention targets. To further our knowledge of the intricate relationships between human health and microbial diversity in the context of CKD, integrated multi-omics research is also necessary. This is a highly prospective and significant future direction.

## Supporting information

S1 Table Characteristics of the study population. Clinical data of hemodialysis patients and baseline information of the two groups.
(PDF)

S2 Table Intake of major nutrients between hemodialysis patients and healthy controls. The average intake of major nutrients in both groups, and there were no significant differences in nutrient intake between the two groups.
(PDF)

S3 Table Fecal identified differential metabolites between hemodialysis patients and healthy controls. Metabolites with significant differences between the two groups and their corresponding metabolic pathways.
(PDF)

S1 Fig The phylum-level taxonomic display of the Hemodialysis patients and Healthy controls. We selected the Top 30 phylums of abundance. The horizontal axis is grouped, and the vertical axis represents the relative abundance of a certain species classification. Different colors correspond to different phylums.
(PDF)

S2 Fig Comparison of the abundance of gut microbiota in hemodialysis patients and healthy controls at the phylum and species level. The figure shows: grouping (left), the abundance of the corresponding phylum level (middle) and genus level (right). Different colors correspond to different species.
(PDF)

S3 Fig Indicator Species Analysis between different groups. The size of the point represents the sqrtIVt value, and the larger the point, the more the ASV can be used as a biomarker. The color represents the *p-*value. sqrtIV: square root result of indicator value, used to compare the correlation between microbiota and treatment groups.
(PDF)

S4 Fig Metabolic patterns in hemodialysis patients and healthy controls. Fecal metabolite profiles in hemodialysis patients and healthy controls were shown as heatmaps. Each row represented data for a differentially expressed metabolite and each column represented an individual. Different colors corresponded to the different relative abundance of metabolites. Red and blue colors represented increased and decreased levels of metabolites, respectively.
(PDF)

S5 Fig Pathway analysis of altered metabolites isolated from HD patients compared with healthy controls. Rich Factor: the number of differential metabolites located in the Pathway / the total number of metabolites contained in the Pathway. The size of the dot represented the number of differential metabolites on the pathway. The color of the dot represented the *p*-value of the enrichment analysis, that is, the significance of the enrichment.
(PDF)

S6 Fig Correlation heatmap of differential species and metabolites between hemodialysis patients and healthy controls. Color represented correlation, red represented positive correlation, and blue represented negative correlation. The darker the color, the stronger the correlation. *: *p* < 0.05，**: *p* < 0.01.
(PDF)

S7 Fig Correlation network of differential species and metabolites between hemodialysis patients and healthy controls. Green triangles represent bacterial genera; purple circles represent metabolites. Solid lines indicate positive Spearman correlations (*p* < 0.05), dashed lines indicate negative Spearman correlations (*p* < 0.05). Only statistically significant correlations are displayed.
(PDF)

S8 Fig Mantel test network heatmap between differential genus and metabolites. The heatmap on the right: the redder the color, the closer the correlation coefficient (r) is to 1. Conversely, the bluer the color, the closer the r-value is to −1. The network diagram in the lower left corner: the color represented the *p*-value, and the thicker the line, the stronger the correlation.
(PDF)

S9 Fig Alpha diversity analysis and Beta diversity analysis between hemodialysis patients and healthy controls. i
(PDF)
