# Supplementary material for: Integrated multi-omics reveals dysbiosis in hemodialysis patients: A multi-center study
Source: PLoS One. 2026 Aug 13;21(8):e0355698. doi: 10.1371/journal.pone.0355698 (PMC13472434; doi:10.1371/journal.pone.0355698)
Supplement: Supplementary file 1 [file pone.0355698.s001.pdf]

Supplementary Table 1: Characteristics of the study population.

	HD	HC
Female (%)	52 (54.2%)	51 (53.1%)
Age, years, median (IQR)	57.5 (46.5, 66.0)	58.0 (43.0, 65.0)
Height, cm, median (IQR)	163.0 (158.0, 170.0)	165.0 (159.3, 172.8)
Weight, kg, median (IQR)	61.4 (53.2, 70.2)	62.5 (56.0, 68.8)
BMI, kg/m^2, median (IQR)	22.5 (20.0, 25.0)	22.4 (21.3, 24.8)
**Disease Parameters**		
KT/V, median (IQR)	1.5 (1.3, 1.7)	
Dialysis duration (months)	43.0 (17.3, 82.5)	
Dialysis access type, n (%)		
Arteriovenous fistula	94 (97.9)	
Central venous cathete	2 (2.1)	
Residual renal function, n (%)		
Yes	81 (85.3)	
**Inflammatory status**		
MIS	5.0 (3.0, 7.0)	
CRP	2.1 (1.1, 4.3)	
NLR	3.6 (2.6, 5.3)	

*BMI body mass index, CRP C-reactive protein, KT/V dialysis adequacy, MIS malnutrition-inflammation score, NLR Neutrophil-lymphocyte Ratio (normal range: 0.78~3.53)
