# Supplementary material for: Integrated multi-omics reveals dysbiosis in hemodialysis patients: A multi-center study
Source: PLoS One. 2026 Aug 13;21(8):e0355698. doi: 10.1371/journal.pone.0355698 (PMC13472434; doi:10.1371/journal.pone.0355698)
Supplement: Supplementary file 2 [file pone.0355698.s002.pdf]

Supplementary Table 2: Intake of major nutrients between hemodialysis patients and healthy controls

Nutrients	Daily intake per person (HD)	Daily intake per person (HC)	*P*-value
Energy(kcal）	1637.22 ± 310.92	1605.78 (1428.31, 1804.36)	0.281
Protein(mg)	59.67 (47.36, 69.40)	63.60 (49.63, 63.39)	0.966
Carbohydrate(mg)	223.20 (181.78, 258.79）	224.51 ± 56.99	0.624
Fat(mg)	58.89 (40.87, 71.58)	58.32 (43.74, 70.45)	0.797
Dietary fiber(mg)	9.64 (6.71, 11.91)	9.73 (6.69, 12.39)	0.783
**Fat-soluble vitamins**			
Vitamin A(μg RAE)	425.00 (170.62 580.31)	427.15 (166.51, 580.15)	0.942
Vitamin E(mg)	20.56(14.15, 26.18)	20.88 (14.66, 26.12)	0.776
**Water-soluble vitamins**			
Vitamin B1(mg)	0.87 (0.66, 1.04)	0.88 ± 0.30	0.622
Vitamin B2(mg)	0.77 (0.57, 0.95)	0.78 (0.60, 0.89)	0.805
Vitamin B3(mg)	10.76 (7.75, 13.11)	10.73 (7.87, 12.62)	0.919
Vitamin C(mg)	67.05 (26.66, 105.40)	67.20 (31.94, 92.98)	0.801
Folic acid(μg)	166.45 (105.40, 203.55)	166.33 (103.15, 200.04)	0.708
**Minerals**			
Potassium(mg)	1433.78 (1150.38, 1713.08)	1435.25 (1063.95, 1736.00)	0.957
Sodium(mg)	3205.85（2368.17，3780.25）	3228.05（2381.25，3786.00）	0.728
Calcium(mg)	388.42 (241.14, 500.65)	390.97 (234.70, 506.80)	0.920
Phosphonium(mg)	859.25 (714.44, 976.90)	858.79 (731.48, 995.70)	0.650

Normally distributed data are described by mean ± standard deviation ($\bar{x}$ ± SD) and skewed distributions are expressed as IQR median (P25, P75).
