# Supplementary material for: Integrated multi-omics reveals dysbiosis in hemodialysis patients: A multi-center study
Source: PLoS One. 2026 Aug 13;21(8):e0355698. doi: 10.1371/journal.pone.0355698 (PMC13472434; doi:10.1371/journal.pone.0355698)
Supplement: Supplementary file 3 [file pone.0355698.s003.pdf]

Supplementary Table 3: Fecal identified differential metabolites between hemodialysis patients andhealthy controls

Metabolite	*P*-value	VIP	FC	Pathways
Imidazoleacetic acid	0.00	5.53	6.78	Amino acid metabolism
4-Pyridoxic acid	0.00	3.36	3.56	Vitamin B6 Metabolism
Ascorbic acid	0.00	3.19	0.30	Vitamin C metabolism
N-Methyl-2-pyridone-5-carbox amide	0.00	3.12	4.09	Vitamin B3 metabolism
Prostaglandin D2	0.00	1.34	0.70	Fatty acid metabolism
PE(P-18:0/18:1(9Z))	0.00	2.20	2.09	GPI−anchor biosynthesis
Dl-Pipecolinic acid	0.00	1.94	0.05	Amino acid metabolism
Biotin	0.00	1.68	0.59	Vitamin B7 Metabolism
D-Xylose	0.00	1.53	0.65	Amino sugar and nucleotide sugar metabolism
5-Methoxyindole-3-acetic acid	0.00	2.07	0.48	Amino acid metabolism
Pimelic acid	0.00	1.50	0.66	Vitamin B7 Metabolism
Adenosine	0.00	2.15	3.32	Purine metabolism
Pyridoxal hydrochrolide	0.00	2.04	2.91	Vitamin B6 Metabolism
L-Aspartate	0.00	1.50	0.64	Amino acid metabolism
D-Arabinose-5-phosphate disodium salt	0.00	1.41	1.56	Lipopolysaccharide metabolism
PG 30:0	0.00	2.51	6.58	Glycerophospholipid metabolism
PG (15:0/16:0)	0.00	2.32	6.54	Glycerophospholipid metabolism
Picolinic acid	0.00	1.01	0.78	Amino acid metabolism
Orotic acid	0.00	1.77	0.49	Pyrimidine metabolism
N-acetyl putrescine hydrochl-oride	0.00	2.01	0.06	Amino acid metabolism

VIP, variable importance in the projection; FC, fold change; GPI: Glycosylphosphatidylinositol; PE, Phosphatidylethanolamine; PG, phosphatidyl glycerol.
