# Supplementary material for: Integrated multi-omics reveals dysbiosis in hemodialysis patients: A multi-center study
Source: PLoS One. 2026 Aug 13;21(8):e0355698. doi: 10.1371/journal.pone.0355698 (PMC13472434; doi:10.1371/journal.pone.0355698)
Supplement: Supplementary file 4 [file pone.0355698.s004.pdf]

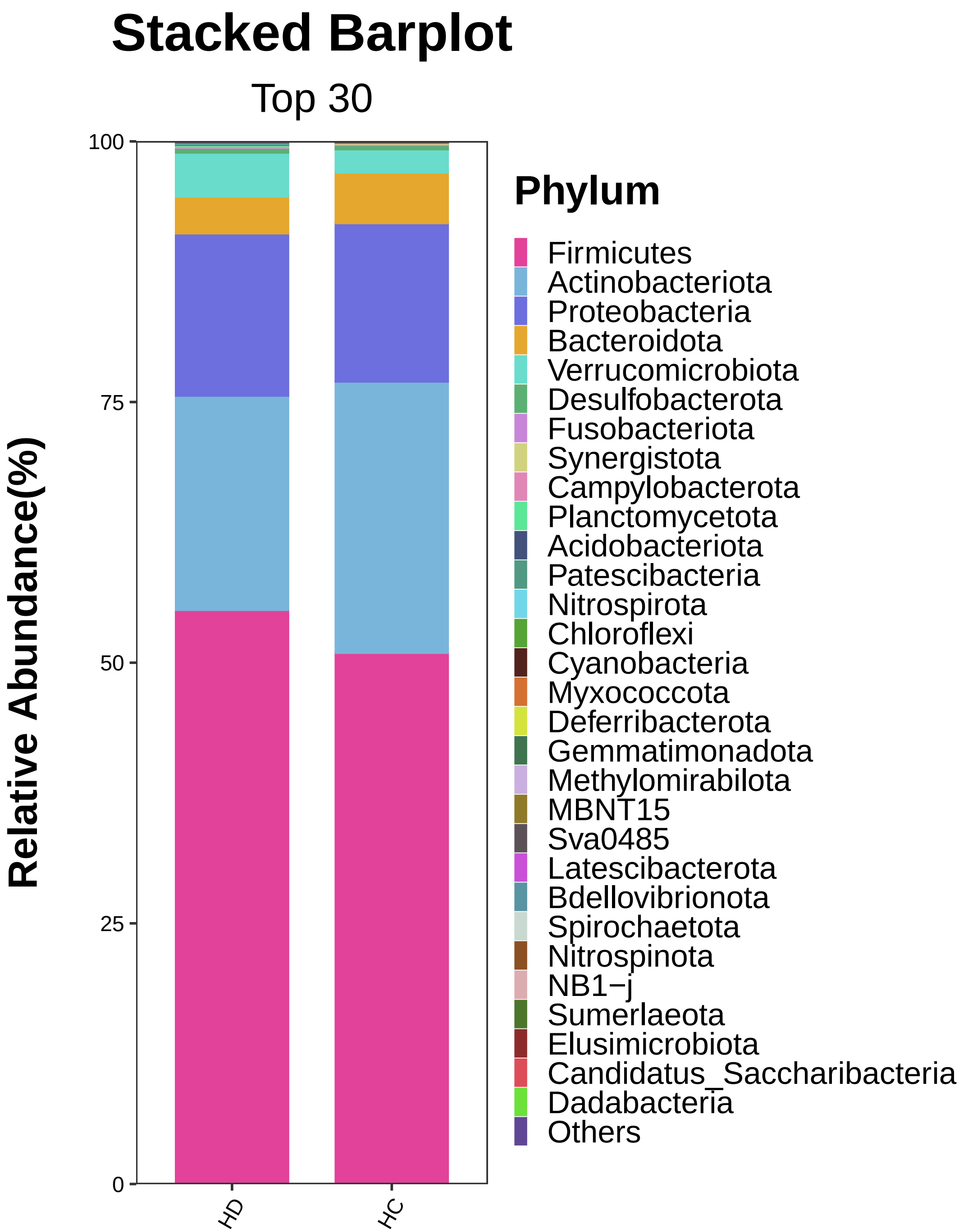
Stacked Barplot
Top 30
Relative Abundance(%)
100
75
50
25
0
HD
HC
Phylum
Firmicutes
Actinobacteriota
Proteobacteria
Bacteroidota
Verrucomicrobiota
Desulfobacterota
Fusobacteriota
Synergistota
Campylobacterota
Planctomycetota
Acidobacteriota
Patescibacteria
Nitrospirota
Chloroflexi
Cyanobacteria
Myxococcota
Deferribacterota
Gemmatimonadota
Methylomirabilota
MBNT15
Sva0485
Latescibacterota
Bdellovibrionota
Spirochaetota
Nitrospinota
NB1−j
Sumerlaeota
Elusimicrobiota
Candidatus_Saccharibacteria
Dadabacteria
Others
