# Supplementary material for: Integrated multi-omics reveals dysbiosis in hemodialysis patients: A multi-center study
Source: PLoS One. 2026 Aug 13;21(8):e0355698. doi: 10.1371/journal.pone.0355698 (PMC13472434; doi:10.1371/journal.pone.0355698)
Supplement: Supplementary file 6 [file pone.0355698.s006.pdf]

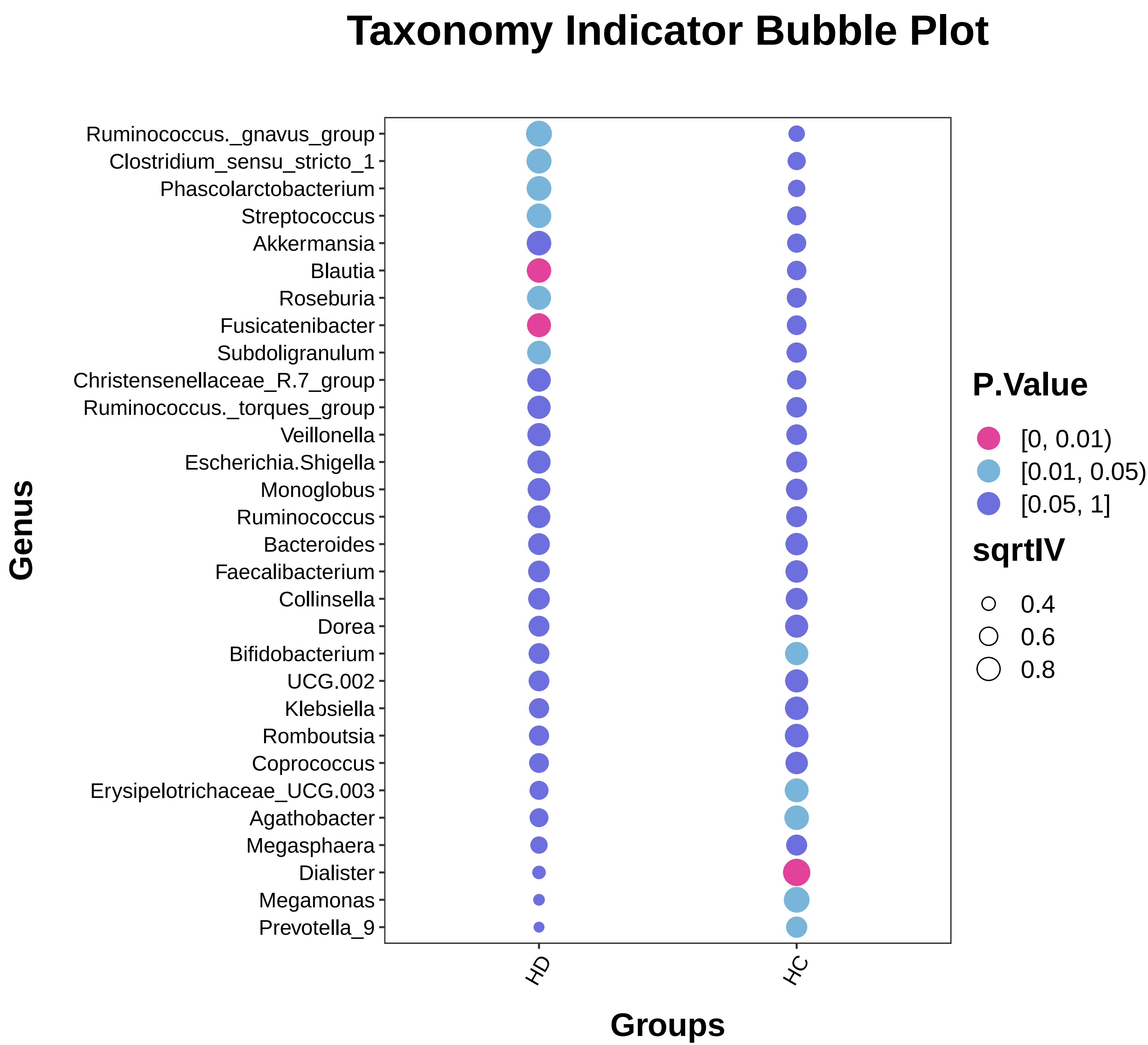
Taxonomy Indicator Bubble Plot
Genus
Ruminococcus._gnavus_group
Clostridium_sensu_stricto_1
Phascolarctobacterium
Streptococcus
Akkermansia
Blautia
Roseburia
Fusicatenibacter
Subdoligranulum
Christensenellaceae_R.7_group
Ruminococcus._torques_group
Veillonella
Escherichia.Shigella
Monoglobus
Ruminococcus
Bacteroides
Faecalibacterium
Collinsella
Dorea
Bifidobacterium
UCG.002
Klebsiella
Romboutsia
Coprococcus
Erysipelotrichaceae_UCG.003
Agathobacter
Megasphaera
Dialister
Megamonas
Prevotella_9
HD
HC
Groups
P.Value
[0, 0.01)
[0.01, 0.05)
[0.05, 1]
sqrtIV
0.4
0.6
0.8
