# Supplementary material for: Integrated multi-omics reveals dysbiosis in hemodialysis patients: A multi-center study
Source: PLoS One. 2026 Aug 13;21(8):e0355698. doi: 10.1371/journal.pone.0355698 (PMC13472434; doi:10.1371/journal.pone.0355698)
Supplement: Supplementary file 7 [file pone.0355698.s007.pdf]

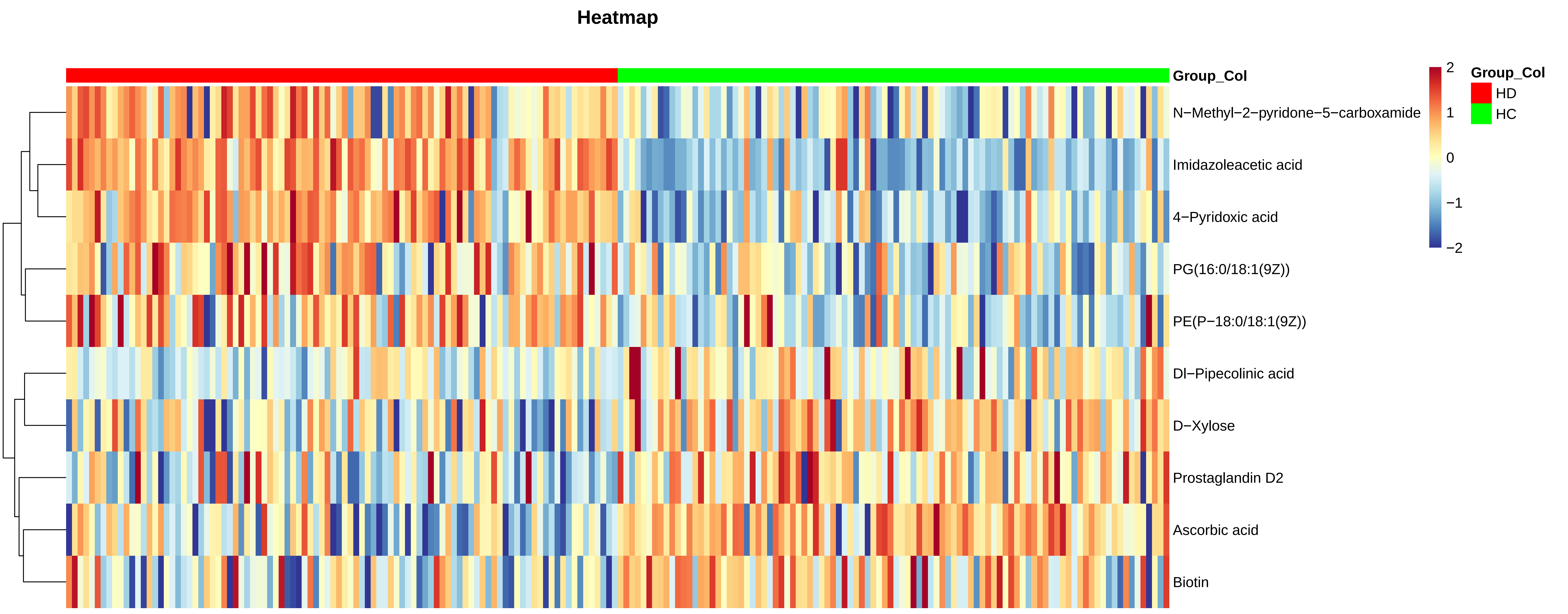
Heatmap
Group_Col
N−Methyl−2−pyridone−5−carboxamide
Imidazoleacetic acid
4−Pyridoxic acid
PG(16:0/18:1(9Z))
PE(P−18:0/18:1(9Z))
Dl−Pipecolinic acid
D−Xylose
Prostaglandin D2
Ascorbic acid
Biotin
2
1
0
−1
−2
Group_Col
HD
HC
