# Supplementary material for: Integrated multi-omics reveals dysbiosis in hemodialysis patients: A multi-center study
Source: PLoS One. 2026 Aug 13;21(8):e0355698. doi: 10.1371/journal.pone.0355698 (PMC13472434; doi:10.1371/journal.pone.0355698)
Supplement: Supplementary file 8 [file pone.0355698.s008.pdf]

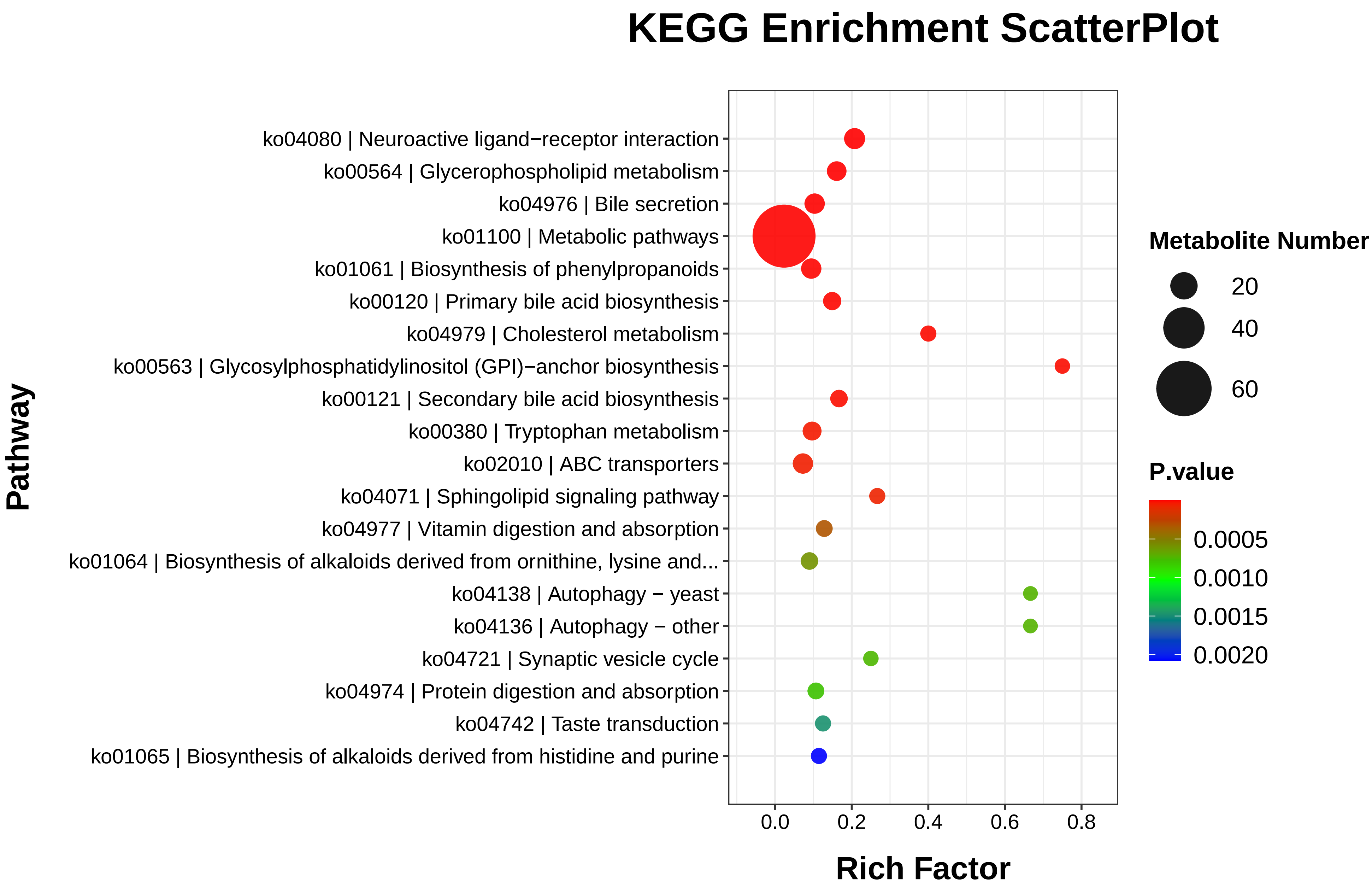
KEGG Enrichment ScatterPlot
ko04080 | Neuroactive ligand−receptor interaction
ko00564 | Glycerophospholipid metabolism
ko04976 | Bile secretion
ko01100 | Metabolic pathways
ko01061 | Biosynthesis of phenylpropanoids
ko00120 | Primary bile acid biosynthesis
ko04979 | Cholesterol metabolism
ko00563 | Glycosylphosphatidylinositol (GPI)−anchor biosynthesis
ko00121 | Secondary bile acid biosynthesis
ko00380 | Tryptophan metabolism
ko02010 | ABC transporters
ko04071 | Sphingolipid signaling pathway
ko04977 | Vitamin digestion and absorption
ko01064 | Biosynthesis of alkaloids derived from ornithine, lysine and...
ko04138 | Autophagy − yeast
ko04136 | Autophagy − other
ko04721 | Synaptic vesicle cycle
ko04974 | Protein digestion and absorption
ko04742 | Taste transduction
ko01065 | Biosynthesis of alkaloids derived from histidine and purine
Pathway
0.0
0.2
0.4
0.6
0.8
Rich Factor
Metabolite Number
20
40
60
P.value
0.0005
0.0010
0.0015
0.0020
