# Supplementary material for: Integrated multi-omics reveals dysbiosis in hemodialysis patients: A multi-center study
Source: PLoS One. 2026 Aug 13;21(8):e0355698. doi: 10.1371/journal.pone.0355698 (PMC13472434; doi:10.1371/journal.pone.0355698)
Supplement: Supplementary file 9 [file pone.0355698.s009.pdf]

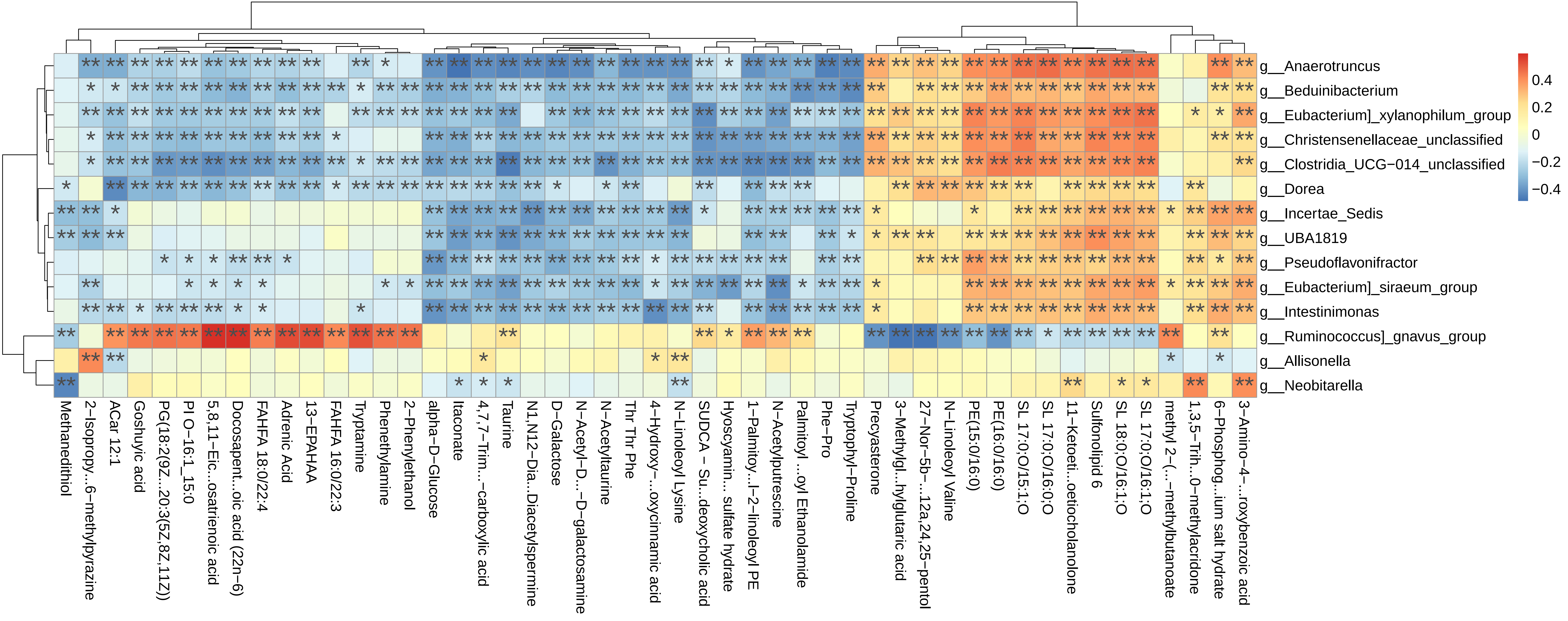

g__Anaerotruncus
g__Beduinibacterium
g__Eubacterium]_xylanophilum_group
g__Christensenellaceae_unclassified
g__Clostridia_UCG−014_unclassified
g__Dorea
g__Incertae_Sedis
g__UBA1819
g__Pseudoflavonifractor
g__Eubacterium]_siraeum_group
g__Intestinimonas
g__Ruminococcus]_gnavus_group
g__Allisonella
g__Neobitarella
0.4
0.2
0
−0.2
−0.4
Methanedithiol
2−Isopropy...6−methylpyrazine
ACar 12:1
Goshuyic acid
PG(18:2(9Z...20:3(5Z,8Z,11Z))
PI O−16:1_15:0
5,8,11−Eic...osatrienoic acid
Docosapent...oic acid (22n−6)
FAHFA 18:0/22:4
Adrenic Acid
13−EPAHAA
FAHFA 16:0/22:3
Tryptamine
Phenethylamine
2−Phenylethanol
alpha−D−Glucose
Itaconate
4,7,7−Trim...−carboxylic acid
Taurine
N1,N12−Dia...Diacetylspermine
D−Galactose
N−Acetyl−D...−D−galactosamine
N−Acetyltaurine
Thr Thr Phe
4−Hydroxy−...oxycinnamic acid
N−Linoleoyl Lysine
SUDCA − Su...deoxycholic acid
Hyoscyamin... sulfate hydrate
1−Palmitoy...l−2−linoleoyl PE
N−Acetylputrescine
Palmitoyl ...oyl Ethanolamide
Phe−Pro
Tryptophyl−Proline
Precyasterone
3−Methylgl...hylglutaric acid
27−Nor−5b−...12a,24,25−pentol
N−Linoleoyl Valine
PE(15:0/16:0)
PE(16:0/16:0)
SL 17:0;O/15:1;O
SL 17:0;O/16:0;O
11−Ketoeti...oetiocholanolone
Sulfonolipid 6
SL 18:0;O/16:1;O
SL 17:0;O/16:1;O
methyl 2−(...−methylbutanoate
1,3,5−Trih...0−methylacridone
6−Phosphog...ium salt hydrate
3−Amino−4−...roxybenzoic acid
