# Supplementary material for: Integrated multi-omics reveals dysbiosis in hemodialysis patients: A multi-center study
Source: PLoS One. 2026 Aug 13;21(8):e0355698. doi: 10.1371/journal.pone.0355698 (PMC13472434; doi:10.1371/journal.pone.0355698)
Supplement: Supplementary file 10 [file pone.0355698.s010.pdf]

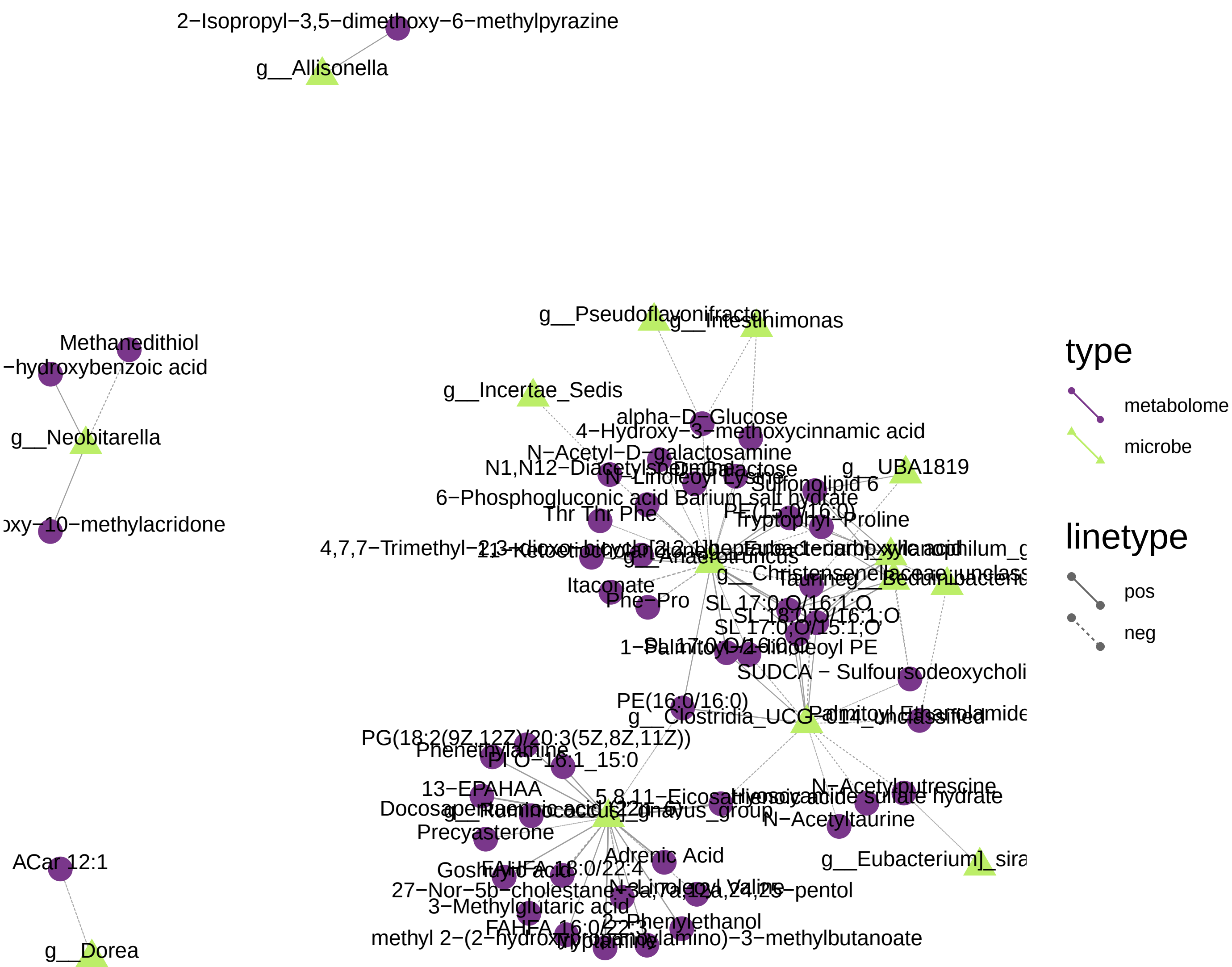

2−Isopropyl−3,5−dimethoxy−6−methylpyrazine
g__Allisonella
Methanedithiol
g__Neobitarella
ACar 12:1
g__Dorea
g__Pseudoflavonifractor
g__Intestinimonas
g__Incertae_Sedis
alpha−D−Glucose
4−Hydroxy−3−methoxycinnamic acid
N−Acetyl−D−galactosamine
g__UBA1819
Sulfonolipid 6
6−Phosphogluconic acid Barium salt hydrate
Thr Thr Phe
Tryptophyl−Proline
Itaconate
Phe−Pro
SL 17:0;O/16:1;O
SL 18:0;O/16:1;O
SL 17:0;O/15:1;O
SUDCA − Sulfoursodeoxycholi
PE(16:0/16:0)
PG(18:2(9Z,12Z)/20:3(5Z,8Z,11Z))
PI O−16:1_15:0
13−EPAHAA
Precyasterone
N−Acetylputrescine
N−Acetyltaurine
g__Eubacterium]_sira
Adrenic Acid
27−Nor−5b−cholestane−3a,7a,12a,24,25−pentol
3−Methylglutaric acid
2−Phenylethanol
methyl 2−(2−hydroxypropanoylamino)−3−methylbutanoate
type
metabolome
microbe
linetype
pos
neg
