# Supplementary material for: Integrated multi-omics reveals dysbiosis in hemodialysis patients: A multi-center study
Source: PLoS One. 2026 Aug 13;21(8):e0355698. doi: 10.1371/journal.pone.0355698 (PMC13472434; doi:10.1371/journal.pone.0355698)
Supplement: Supplementary file 11 [file pone.0355698.s011.pdf]

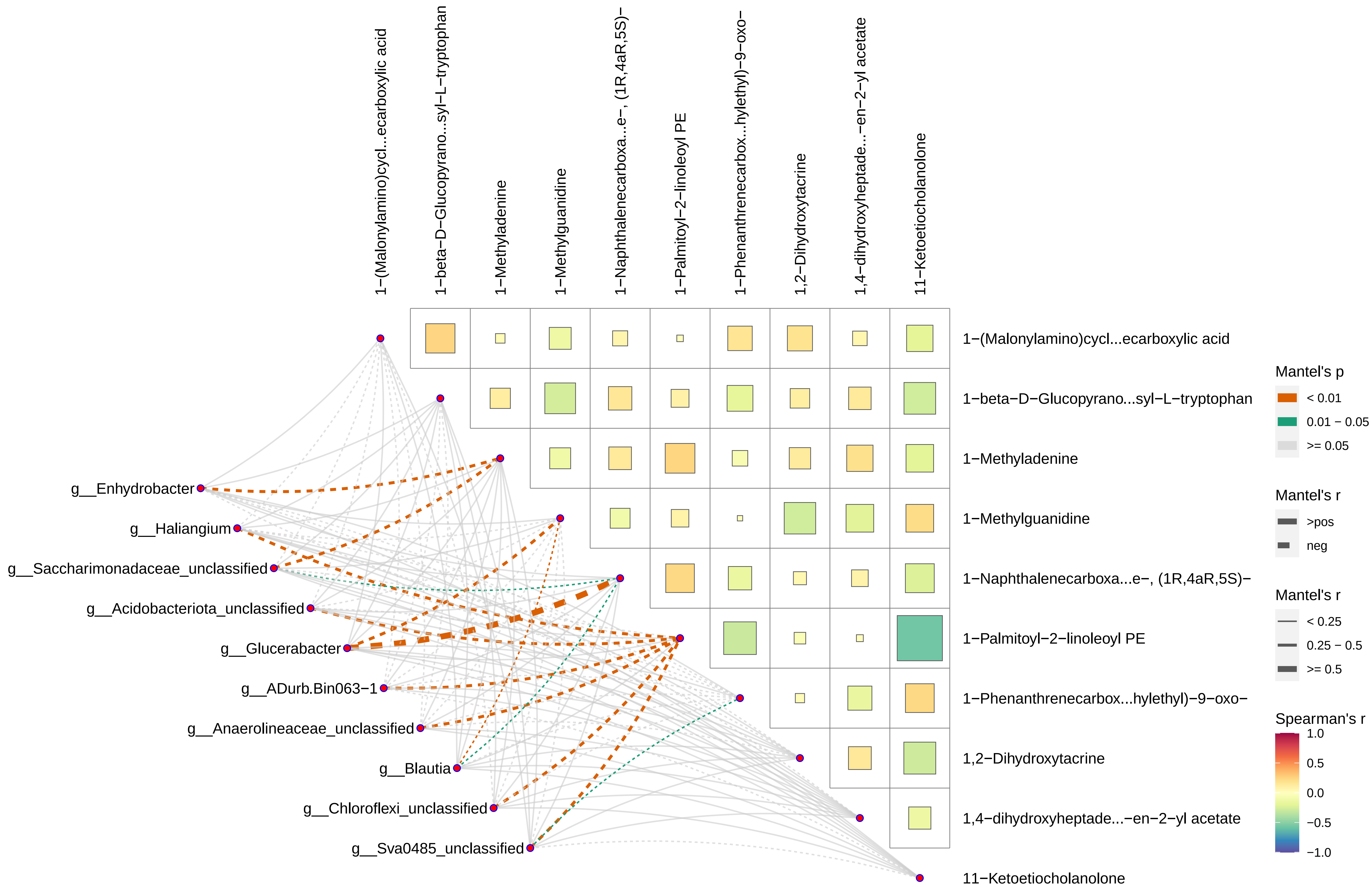

1−(Malonylamino)cycl...ecarboxylic acid
1−beta−D−Glucopyrano...syl−L−tryptophan
1−Methyladenine
1−Methylguanidine
1−Naphthalenecarboxa...e−, (1R,4aR,5S)−
1−Palmitoyl−2−linoleoyl PE
1−Phenanthrenecarbox...hylethyl)−9−oxo−
1,2−Dihydroxytacrine
1,4−dihydroxyheptade...−en−2−yl acetate
11−Ketoetiocholanolone
g__Enhydrobacter
g__Haliangium
g__Saccharimonadaceae_unclassified
g__Acidobacteriota_unclassified
g__Glucerabacter
g__ADurb.Bin063−1
g__Anaerolineaceae_unclassified
g__Blautia
g__Chloroflexi_unclassified
g__Sva0485_unclassified
Mantel's p
< 0.01
0.01 − 0.05
>= 0.05
Mantel's r
>pos
neg
Mantel's r
< 0.25
0.25 − 0.5
>= 0.5
Spearman's r
1.0
0.5
0.0
−0.5
−1.0
