# Supplementary material for: Integrated multi-omics reveals dysbiosis in hemodialysis patients: A multi-center study
Source: PLoS One. 2026 Aug 13;21(8):e0355698. doi: 10.1371/journal.pone.0355698 (PMC13472434; doi:10.1371/journal.pone.0355698)
Supplement: Supplementary file 12 [file pone.0355698.s012.pdf]

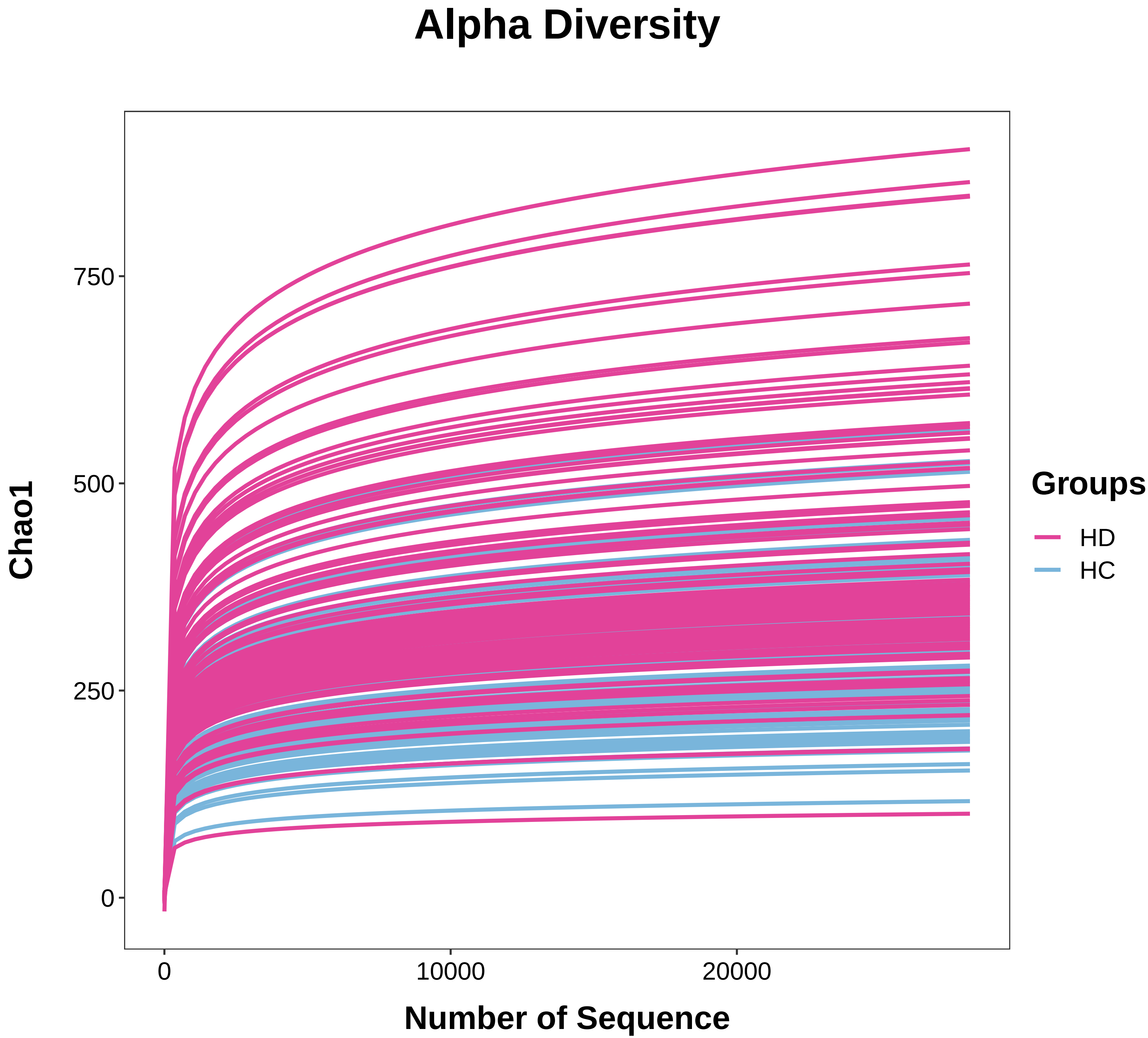
Alpha Diversity
Chao1
750
500
250
0
0
10000
20000
Number of Sequence
Groups
HD
HC

Alpha Diversity

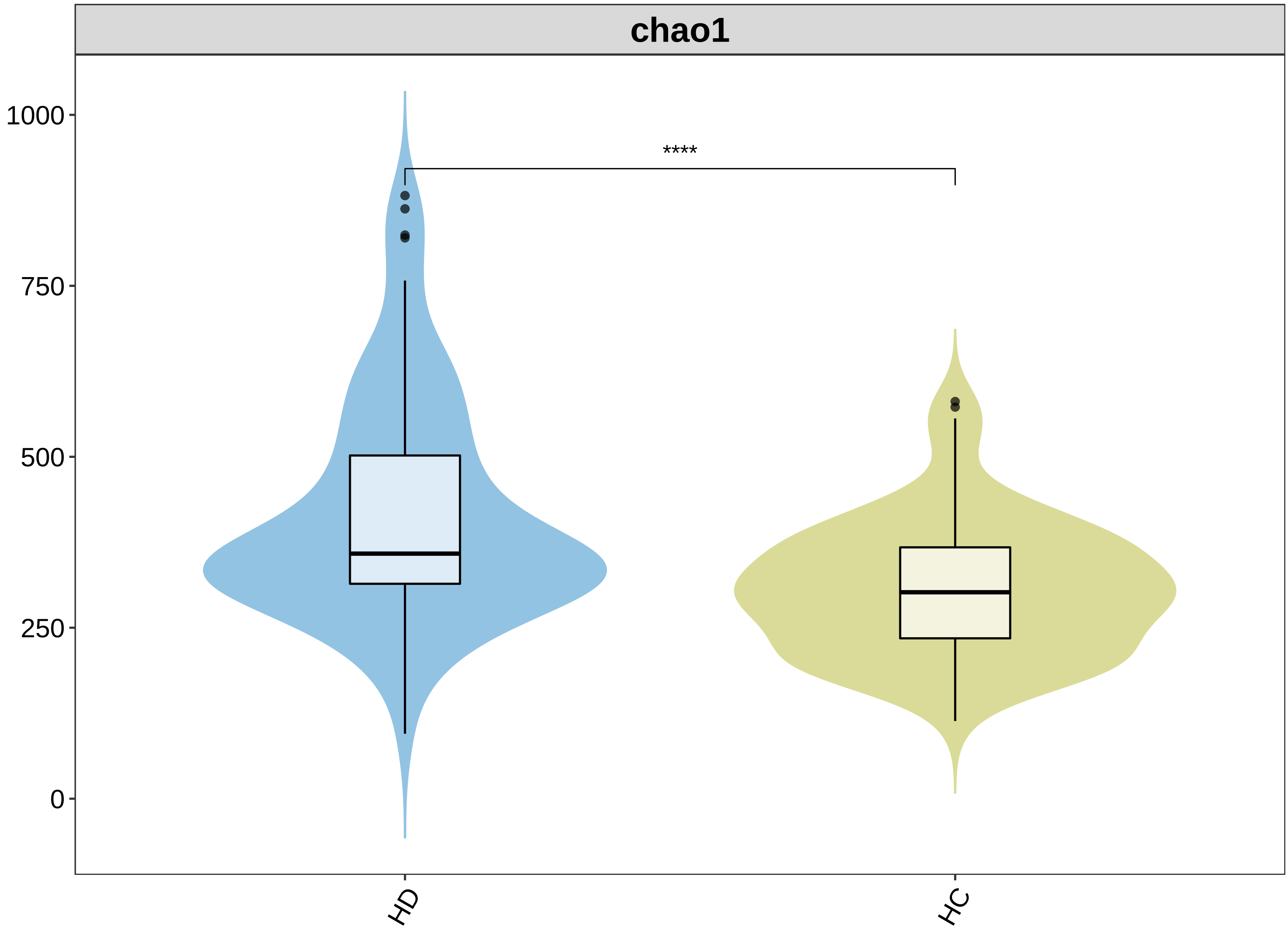

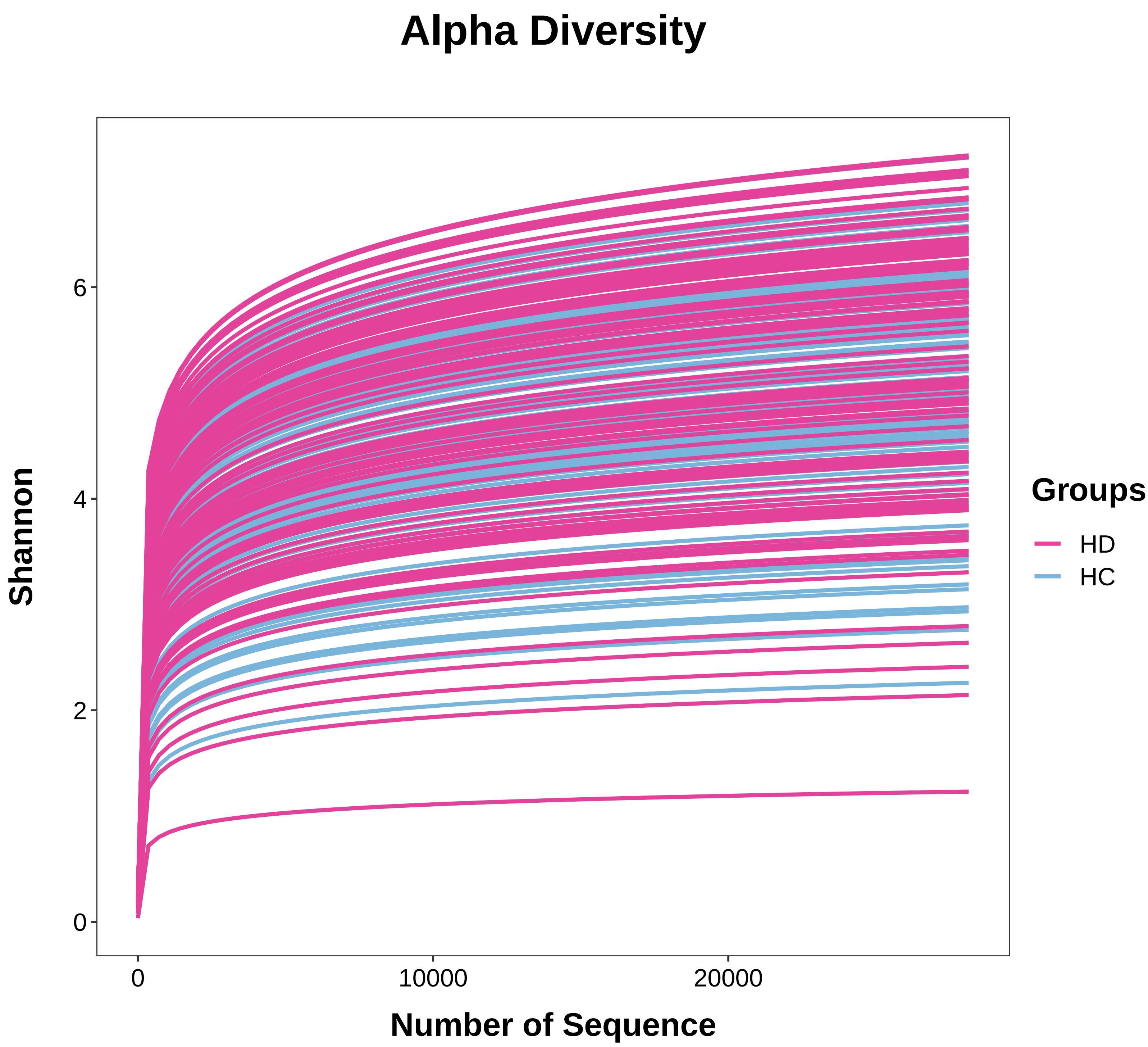
Alpha Diversity
Shannon
Number of Sequence
0
2
4
6
0
10000
20000
Groups
HD
HC

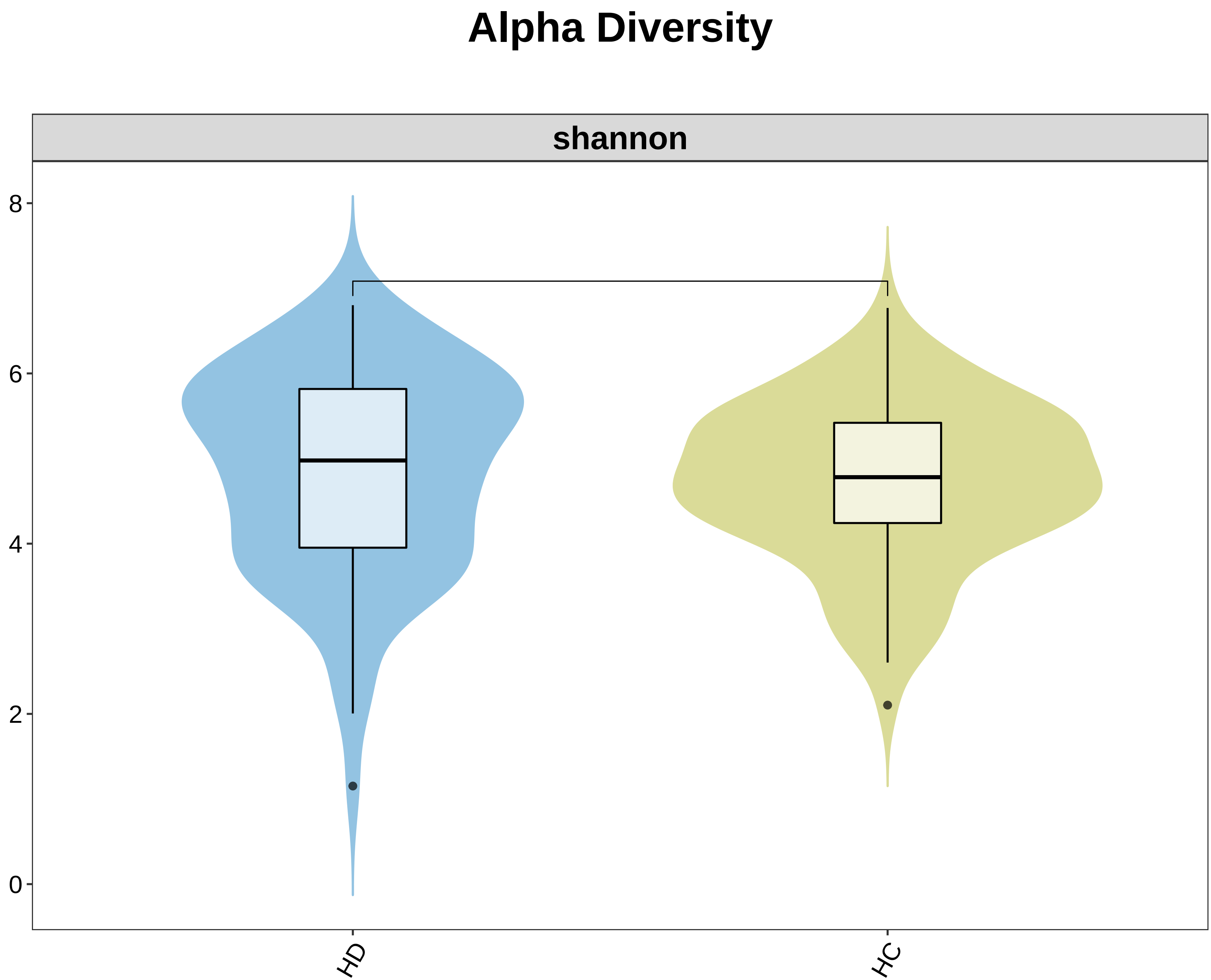

Alpha Diversity
shannon
8
6
4
2
0
HD
HC

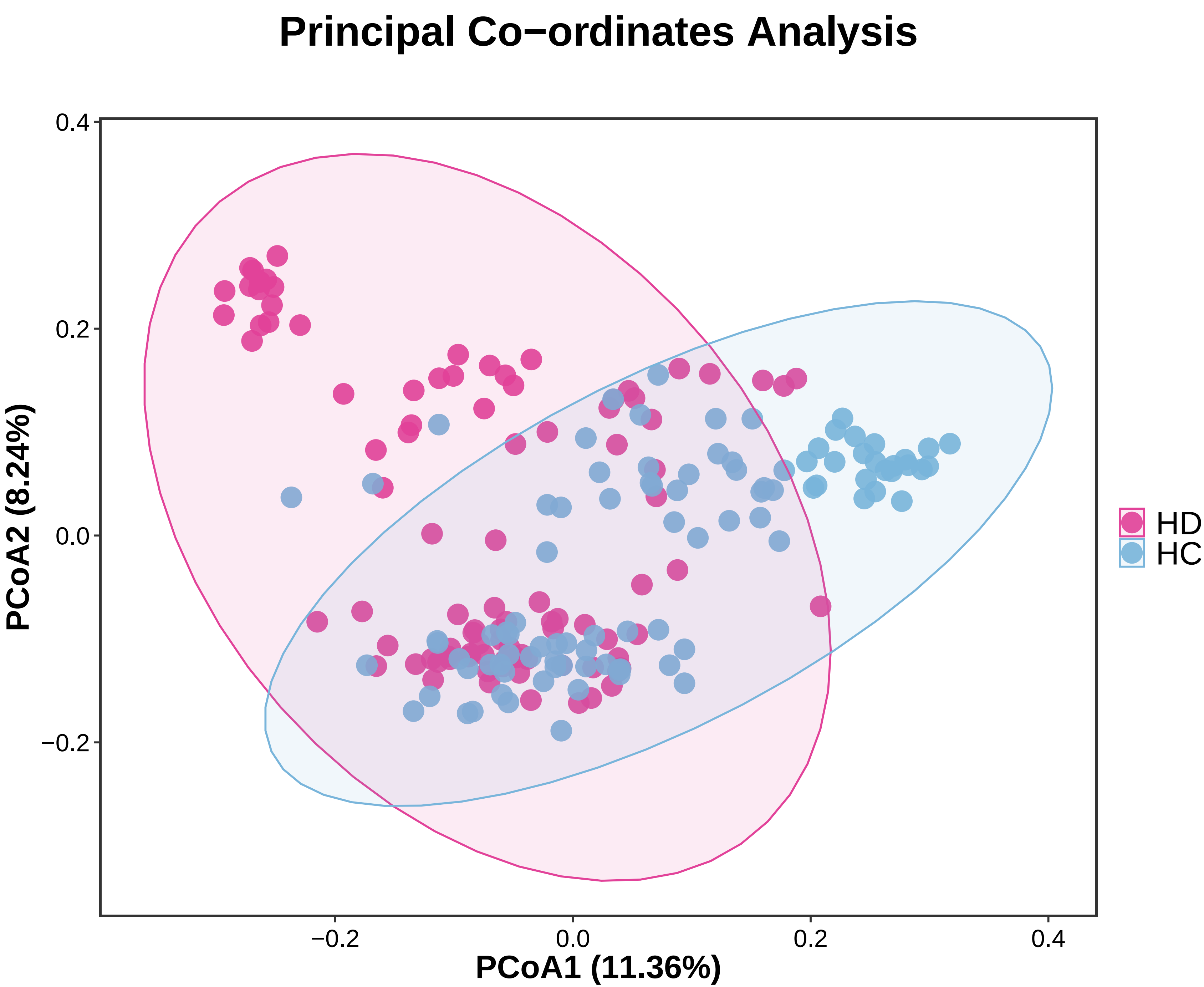
Principal Co−ordinates Analysis
PCoA2 (8.24%)
PCoA1 (11.36%)
0.4
0.2
0.0
−0.2
−0.2
0.0
0.2
0.4
HD
HC

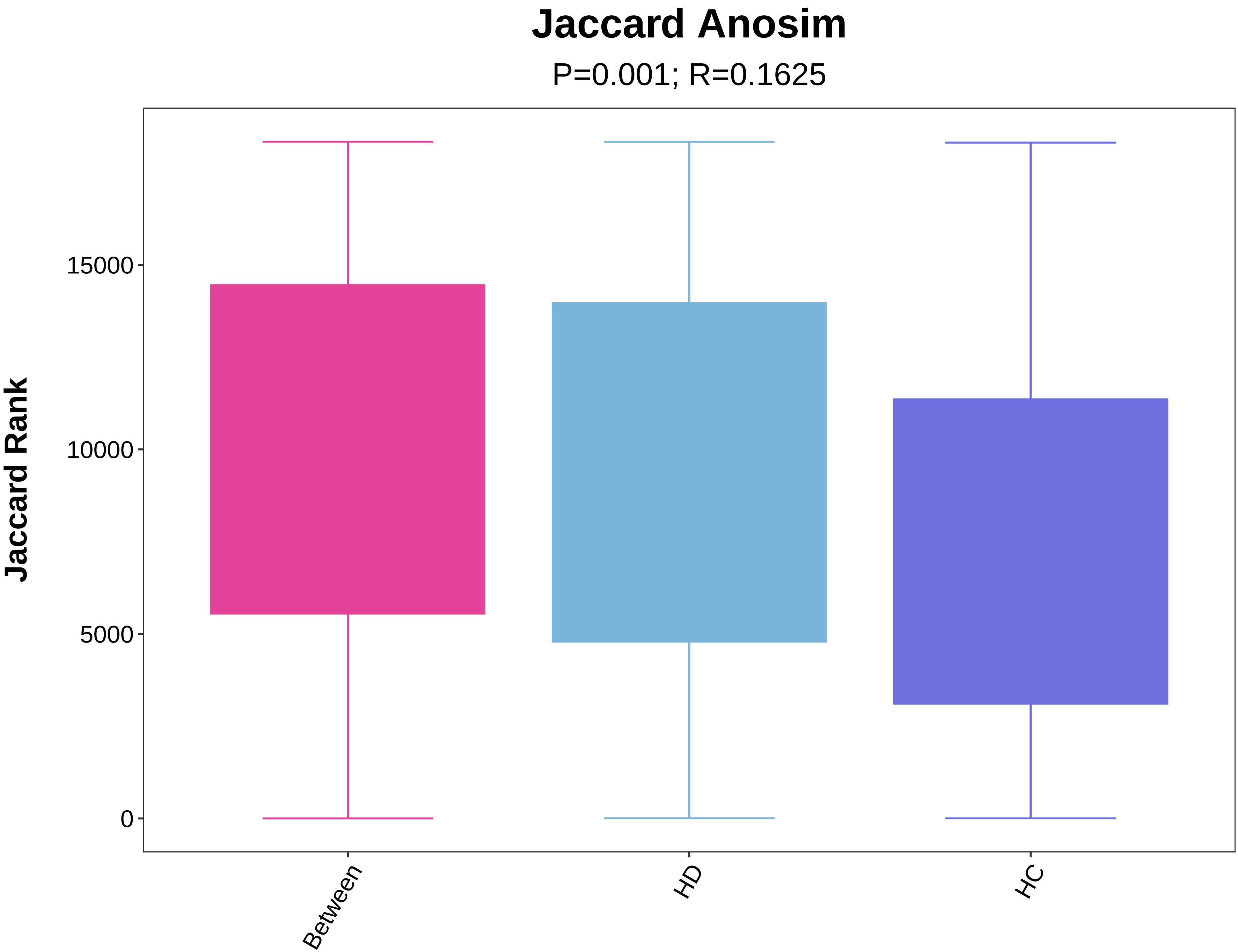
Jaccard Anosim
P=0.001; R=0.1625
Jaccard Rank
15000
10000
5000
0
Between
HD
HC
