# Supplementary material for: Integrated multi-omics reveals dysbiosis in hemodialysis patients: A multi-center study
Source: PLoS One. 2026 Aug 13;21(8):e0355698. doi: 10.1371/journal.pone.0355698 (PMC13472434; doi:10.1371/journal.pone.0355698)
Supplement: Supplementary file 13 [file pone.0355698.s014.docx]

**Response Letter**

Manuscript Title: [Integrated Multi-Omics Reveals Dysbiosis in Hemodialysis Patients: A Multi-Center Study]

Journal Name: [PLOS One]

Manuscript ID: [PONE-D-25-56310]

Dear Editor and Reviewers:

A pleasant day to you!

We sincerely thank you for the opportunity to revise our manuscript and for the constructive comments provided by the reviewers. We have carefully addressed all major concerns raised by Reviewers 2, 3, and 4. For initially unclear points, we consulted academic advisors and field professionals to ensure accurate understanding and proper responses. We believe the revised manuscript now meets the standards for publication in PLOS ONE.

We have formatted the revised manuscript according to the PLOS ONE templates, including proper file naming, line numbering, title page, and figure formatting. We have corrected the funding information in the online submission system. The grant number is now consistent: No. ZF2024069. We have regenerated and uploaded high-resolution versions of all requested figures. Clarity has been substantially improved. We have addressed all editorial requirements as follows: no custom code was generated (SPSS/GraphPad Prism used); the funding statement has been amended to include “*There was no additional external funding received for this study”*; the funders had no role in the study; and all data underlying the findings are fully available within the manuscript and its Supporting Information files. Raw sequencing data were uploaded to a public database to meet transparency requirements. DOI: 10.57760/sciencedb.34128. These updates have been detailed in our revised cover letter.

Below we provide a point-by-point response to each reviewer comment. All changes in the revised manuscript have been highlighted for your convenience.

Thank you once again for your time and valuable input. We look forward to your response.

Sincerely,

Yours,

Hailing Di

**Editor Comments**

This manuscript employs an integrated multi-omics approach combining 16S rRNA gene sequencing and untargeted fecal metabolomics to conduct a 1:1 matched case-control study investigating the characteristics of gut dysbiosis in hemodialysis (HD) patients, with HD patients and cohabiting healthy controls (HC) sharing identical dietary and living conditions enrolled as the study subjects. The clinical question addressed by this study is of critical importance: the crosstalk between the gut microbiome and metabolome is closely associated with adverse clinical outcomes in HD patients, and the paired design adopted in this study effectively controls for diet, the most prominent confounding factor in gut microbiome research. The study has a relatively sufficient sample size (96 HD-HC matched pairs) and a rigorous multi-omics analytical workflow. A total of 4 peer reviewers were invited to evaluate this manuscript during the review process. Reviewer 1 highly recognized this study, stating that the work has a rigorous design, reliable multi-omics methodology, and promising findings regarding the identified biomarkers, and recommended direct acceptance without revisions. However, Reviewers 2, 3, and 4 all raised multiple critical concerns, covering fundamental errors in epidemiological data and calculation formulas, inconsistent descriptions of the study design, insufficient methodological details, logical inconsistency between the introduction and study results, inadequate elaboration of innovation and clinical translation value, missing data visualization figures, and incomplete disclosure of study limitations, among other aspects. As the Academic Editor handling this manuscript, I have comprehensively evaluated the full text of the manuscript and all reviewer comments. I confirm that this study has scientific value and clinical relevance, but there are major flaws in academic rigor, methodological accuracy, and content completeness, which must be fully addressed through a major revision.

Response: We sincerely thank you for your thorough evaluation of our manuscript and for your thoughtful summary of the reviewers’ comments. We greatly appreciate the time and effort you and the reviewers have dedicated to improving our work. In response, we have carefully addressed all major concerns raised by Reviewers 2, 3, and 4 through substantial revisions, including corrections to epidemiological data, clarification of the study design, improvement of methodological transparency, restructuring of the introduction and conclusion, addition of required figures, and full disclosure of study limitations. We believe the revised manuscript now meets the standards of academic rigor and completeness required for publication in PLOS ONE. Thank you once again for your guidance. We look forward to your further consideration. **Kind reminder:** If you notice any discrepancies between the line numbers and the content in the revised manuscript, this may be due to differences between word processing software versions (e.g., Word/WPS). Please refer to the PDF version of the manuscript for accurate alignment. Thank you very much for your understanding.

**Reviewers’ Comments**

**Comment 1:**

1. Correction of the Accuracy of Core Data and Epidemiological Statements

(1) Correction of prevalence data for hemodialysis patients

The statement in the manuscript that “the number of HD patients in China had reached 8.44 million by the end of December 2022" has a fundamental deviation from authoritative epidemiological data. As pointed out by Reviewer 3, this figure corresponds to the total prevalence of chronic kidney disease (CKD) in China, not the number of patients receiving maintenance hemodialysis. The authors must recheck the data from authoritative sources, including the China Kidney Disease Network (CK-NET) Annual Report and the National Medical Quality Report on Kidney Diseases issued by the National Health Commission of the People’s Republic of China, revise this figure, clearly distinguish between the total CKD population and maintenance hemodialysis patient population, and provide valid peer-reviewed references to support the revised data.

Response: We thank the reviewers for these insightful observations. We agree and have addressed them in the revised manuscript. The specific corresponding revisions are as follows: We apologize for the previous mistake. The necessary corrections and updates have been made. We have revised the data based on the most recent official statistics. The relevant statements have been revised and clarified as follows: *“According to the latest data from the China National Renal Data System (CNRDS), HD constituted approximately 86.8% of the dialysis population in China, and the number of patients receiving maintenance hemodialysis reached 1,027,267 by the end of 2024”*. This data was officially presented by Academician Chen Xiangmei at the 19th Conference on Critical Kidney Disease and Blood Purification of the Chinese Society of Nephrology in July 2025**^[1]^**. **(Page 4, Line 67-68)**

We have carefully checked the CK NET 2017-2018 Annual Data Report, published in Kidney International Supplements in 2025, and the quality reports from the National Health Commission**^[2]^**. The CK NET report gives useful information on dialysis prevalence, 384.41 per million population in 2017, but its data come from 2017 to 2018. That is not the most current source for patient numbers. The National Health Commission quality reports focus more on process measures and infrastructure, such as the coverage of dialysis services, rather than total patient counts. Therefore, for the most recent national estimate of hemodialysis patients in China, we used the CNRDS data presented by Academician Chen Xiangmei at the 2025 Congress of the Chinese Society of Nephrology. That report showed 1,027,267 hemodialysis patients as of December 2024. We have also cited the CK NET report as supporting evidence for disease burden and dialysis prevalence patterns.

(2) Correction of the overgeneralized statement on renal replacement therapy modalities

The claim in the manuscript that "hemodialysis accounts for nearly 90% of patients receiving renal replacement therapy in most countries" is inaccurate and overly generalized, as the distribution of dialysis modalities varies significantly across countries, regions, and healthcare systems. The authors must revise this statement, provide region-specific data supported by appropriate references, or add rigorous qualifiers to avoid overgeneralization.

Response: We thank the reviewer for this important methodological critique. We agree that the original statement was overly generalized and lacked supporting regional data. We have substantially revised this section as follows: *“Globally, hemodialysis (HD) is the predominant modality, used by approximately 85% of dialysis patients, whereas peritoneal dialysis (PD) accounts for about 11%, with substantial variation across countries. According to the latest data from the China National Renal Data System (CNRDS), HD constituted approximately 86.8% of the dialysis population in China”.*

(1) Global-level data: We have replaced the vague claim with specific percentages from authoritative 2023-2024 sources: (a) In the Apollo Dial DB analysis of 543,169 patients across 40 countries (JASN 2024), 84.8% used HD and 11.0% used PD. Both sources converge on HD accounting for approximately 85% of the global dialysis population^[3]^; (b) Okpechi and colleagues published a cross sectional survey of 148 countries in BMJ in 2024^[4]^ (CAS Q1, IF 43). The survey confirms that hemodialysis is the dominant form of kidney replacement therapy worldwide. A companion paper shows that access to in center HD far exceeds access to PD. Between 2019 and 2023, the number of HD centers increased from 4.4 to 4.8 per million population;
(2) Recognition of regional variation: We have added explicit qualifiers acknowledging substantial inter-country and inter-regional differences, with the understanding that these global averages mask significant heterogeneity.
(3) China-specific data: We have incorporated the latest CNRDS data (December 2024), showing 1,027,267 HD patients and 156,000 PD patients, with HD constituting approximately 86.8% of the dialysis population in mainland China.

We believe these revisions appropriately address the reviewer’s concern by providing specific, referenced data and acknowledging regional variations rather than making a generalized claim. Manuscript. **(Page 4, Line 63-67)**

(3) Correction of errors in key calculation and statistical formulas

The reversed BMI calculation formula on Line 139 of the manuscript is a serious and unacceptable error in epidemiological research. The authors must immediately correct the formula to: Body Mass Index (BMI) = weight (kg) / height² (m²). In addition, as noted by Reviewer 2, the authors must conduct a comprehensive review of all statistical formulas, descriptions of fold-change (FC) thresholds, and screening criteria for differential features in the manuscript, correct all errors, and ensure 100% consistency between the methodological description and the actual analytical workflow.]

**1.3 Response:** We thank the reviewer for their careful review. We have corrected the BMI formula: “*BMI = weight (kg) / [height (m)]²*”. Meanwhile, we have rechecked all BMI calculations and ensured that every computation in the manuscript was performed using the correct formula. No actual data or analyses were affected. We thank the reviewer for this rigorous and constructive comment;

We have conducted a line-by-line review of all statistical formulas, FC thresholds, and screening criteria in the manuscript, cross-checking every detail against our original analysis scripts (*R* code) and software parameters. The following corrections and clarifications have been made:

(1) Fold-change definition -We have explicitly defined FC as mean abundance in the HD group divided by mean abundance in the HC group at the Metabolomics data processing and statistical analysis section. We have substantially revised this section as follows: *“FC was calculated as mean abundance in the HD group divided by mean abundance in the HC group*”**(Page 10, Line 233-234)**
(2) Differential metabolite screening criteria -The original description contained inconsistent thresholds (e.g., “FC ≤ 1.2 or FC ≤ 1.2” and missing down-regulation threshold). 4)Correlation analysis metabolite criteria-The same error (FC ≤ 1.2) has been corrected to FC ≤ 0.833. We have revised the criteria to:

*Absolute FC ≥ 1.2 (FC ≥ 1.2 for up-regulated metabolites or FC ≤ 0.833 for down-regulated metabolites )*;

*Paired samples t-test *p* < 0.05 (on log2-transformed abundances)*;

*VIP ≥ 1 from PLS-DA*. **(Page 10, Line 231-233, and Line246, Line313)**

(3) The Benjamini-Hochberg FDR adjustment was calculated but used only for descriptive purposes; the primary screening used nominal *p* < 0.05 (this is now clearly stated). We have substantially revised this section as follows: *“A nominal p < 0.05 (paired samples t-test) was used as the initial threshold, and Benjamini-Hochberg FDR-adjusted p-values were calculated for multiple testing but not used as a strict inclusion criterion*”**(Page 10, Line 234-236)**
(4) Multiple testing correction in microbiome analysis – We thank the reviewer for pointing out the lack of clarity regarding multiple comparison correction in the 16S data analysis. In fact, the Benjamini-Hochberg false discovery rate (FDR) correction was already applied to the Wilcoxon signed-rank test results for differential genus identification. However, this information was inadvertently omitted from the Methods section, leading to confusion. We sincerely apologize for this oversight. The Methods section has now been revised to explicitly state: “Differential genera were identified using the Wilcoxon signed-rank test with Benjamini-Hochberg FDR correction; a genus was considered significantly different if FDR-adjusted *p* < 0.05.” No changes to the actual data or results were needed, as the correction had been performed from the beginning. We have also verified that all reported differential genera satisfy FDR < 0.05. We have substantially revised this section as follows: *“The differentially abundant genera were identified using the Wilcoxon signed-rank test with Benjamini-Hochberg false discovery rate (FDR) correction. A genus was considered significantly different if FDR-adjusted p < 0.05*”**(Page 9, Line 191-193)**

(5) Consistency verification-We have compared every methodological statement against the actual R scripts and software settings (LC-Bio’s standard pipeline for 16S, XCMS/metaX for metabolomics). All parameters (e.g., VIP cutoff 1.0, t-test type paired, log2 transformation, FC thresholds) now match exactly. We have also prepared a Table that summarizes all analytical thresholds, statistical tests, and correction methods for transparency.

| **Analysis step** | | **Statistical test** | | **Threshold(s)** | **Multiple correction** | |
| --- | --- | --- | --- | --- | --- | --- |
| Differential genera | Wilcoxon signed-rank test | | FDR-adjusted *p* < 0.05 | | | Benjamini-Hochberg |
| Differential metabolites | Paired t-test (log2) | | *p* < 0.05, \|FC\| ≥ 1.2, VIP ≥ 1 | | | FDR calculated but not used as cutoff |
| Correlation (Spearman) | Spearman’s rank | | *p* < 0.05 (uncorrected) | | | None |

All revised text is highlighted in the manuscript. We believe the methodology is now fully accurate and consistent with the executed analysis. We thank the reviewer again for raising the above points. After careful revision and thorough verification, we are confident that the scientific rigor of the manuscript has been substantially improved. If any further concerns remain, we would greatly appreciate your guidance and will make every effort to address them promptly.

**Comment 2:**

Resolution of Core Issues in Study Design and Methodology

(1) Elimination of contradictory descriptions of the study type

The manuscript simultaneously describes the study as both a "prospectively recruited matched case-control study" and a "retrospective study", which constitutes a fundamental contradiction that directly affects the level of evidence and ethical compliance of the study. The authors must explicitly define the study type:

If it is a retrospective study: Supplement full details of the retrospective design, the time frame of data collection, and the approval status of the waiver of informed consent by the ethics committee (if applicable).

If it is a prospective case-control study: Delete all references to "retrospective study" in the manuscript, supplement full details of the prospective recruitment process and the implementation of inclusion and exclusion criteria, and ensure completely consistent description of the study type throughout the manuscript.

Response: We sincerely apologize for the confusion caused by the inconsistent description of the study design. This study is prospective in nature, and we have consistently revised all relevant content throughout the manuscript accordingly. All participants (both hemodialysis patients and healthy controls) were prospectively enrolled between January 2024 and January 2025. Biological samples and clinical data were collected at the time of enrollment, and written informed consent was obtained from every participant before any study procedure. **(Page 6, Line 106-112)**. We have now thoroughly revised the Materials and Methods section to uniformly describe the study as a prospective 1:1 matched case-control study. Specifically, we have:

(1) Explicitly stated the prospective enrollment and data collection timeframe (July 2023- April 2024); **(Page 6, Line 109-110)**
(2) Clearly listed inclusion and exclusion criteria for both HD patients and healthy controls; **(Page 6, Line 122-133)**
(3) Clarified that written informed consent was obtained before enrollment; **(Page 6, Line 111-114)**
(4) Removed any wording that implied a retrospective or previously published cohort.

All revised text is highlighted in the manuscript (Materials and Methods section). We have also checked the abstract, results, discussion, and ethical approval sections to ensure consistent wording. We regret this error and thank the reviewer for the opportunity to correct it.

(2) Supplementation of details on the matching strategy and confounding factor control

As pointed out by Reviewer 3, the description of the 1:1 matching strategy is overly vague. The authors must supplement the following content in the Materials and Methods section:

Additional matching variables beyond age (±2 years) and sex, as well as the rationale for selecting the paired design;The rationale for selecting paired statistical tests (paired t-test / Wilcoxon signed-rank test), and the results of statistical power verification for the paired design;

The specific method of dietary assessment (e.g., 3-day 24-hour dietary recall method, food frequency questionnaire), the time frame of dietary monitoring, and the statistical results verifying dietary homogeneity between cohabiting HD-HC pairs, to validate the core advantage of the study design and address the reviewers’ concerns about the effectiveness of dietary control.

Response: We thank the reviewer for this comment. We apologize for the previous vague description.

(1) Additional matching variables and rationale for the paired design

Considering that diet, geographic water sources, and living environment have a decisive impact on gut microbiota^[5-7]^ (Studies by Rothschild D, Zhou X, and Li C all demonstrated the necessity of tightly controlling for diet and cohabitation factors), We adopt the paired design, each HD patient was paired with a healthy control from the same family, thereby minimizing the impact of these factors. The cohabiting pairs shared dietary sources, environmental exposures, and daily routines. This design allowed us to control more effectively for the key confounding effects of diet and cohabitation on gut microbiota; it's thus methodologically superior to unmatched case-control designs. However, due to the cohabiting pair recruitment condition (the control could be an adult child, spouse, parent, or other cohabiting family member), we were unable to strictly match for age and sex. Detailed baseline characteristics are presented in Supplementary Table 1.

Why we used paired statistical tests. Because each HC is paired with a specific HD patient from the same household, the two groups are not independent. We therefore used the paired t test or Wilcoxon signed rank test, which are the correct choices for matched pair designs and increase statistical power. We have substantially revised this section as follows: *“Based on our household paired design, the Paired Samples t-test (for normally distributed data) and the Wilcoxon signed-rank test (for non‑normally distributed data) were employed”.***(Page 11, Line 256-258)**

We have revised the Materials and Methods section and Supplementary Table 1 accordingly. We apologize for the earlier confusion and thank the reviewer for helping us clarify this. **(Page 6, Line 115-121)**

**(2)** Rationale for paired statistical tests and power verification

We have added a justification for our choice of paired statistical tests. Given the 1:1 household matching design, each HD patient was paired with a cohabiting HC, creating a natural pair structure. For normally distributed variables (identified via Shapiro-Wilk test), we applied the paired t-test; for non-normally distributed variables, we used the Wilcoxon signed-rank test. These paired tests are appropriate because they account for the inherent correlation between matched pairs, thereby reducing within-pair variance and increasing statistical efficiency relative to unpaired tests.

Regarding statistical power: our sample size of 96 matched pairs was determined based on pilot data from our preliminary experiments. Using a paired design, the detectable effect size is approximately equivalent to that of an unmatched design with substantially larger sample size, as paired comparisons eliminate between-pair variance. To formally verify power, we performed post-hoc power calculations using the web-based tool described by Mattiello et al. (Bioinformatics) for case-control microbiome studies**^[8]^**. With n = 96 matched pairs, a two-sided alpha = 0.05, and assuming moderate effect sizes (Cohen’ s d = 0.4-0.5) commonly observed in differential abundance analyses, our study achieved >85% power to detect the observed differences in gut microbial diversity and differential metabolites. This information has been added to the Statistical analyses subsection. We have substantially revised this section as follows: *“Statistical power verification Post-hoc power analysis was performed using the web-based tool for case-control microbiome studies. With a sample size of 96 matched pairs, a two-sided alpha of 0.05, and moderate effect sizes (Cohen’ s d = 0.4-0.5), our study achieved > 85% power to detect the observed differences in gut microbial diversity and differential metabolites”.***(Page 11, Line 258-261)**

**(3)** Dietary assessment method and verification of dietary homogeneity

We agree with the reviewer that dietary homogeneity between HD-HC pairs is the core advantage of our household paired design and requires rigorous verification. We have therefore added detailed information on our dietary assessment approach. We have substantially revised the section *“Dietary intake survey and evaluation”* **(Page 7, Line 134-141)**

1) Assessment method: Dietary intake was assessed using a validated 3-day 24-hour dietary recall method, administered by trained dietitians through face-to-face interviews. This timeframe (3 days) is standard for short-term dietary intake assessment in nutritional epidemiology and is recommended by the MetaCardis study-a large European multi-center gut microbiota study-which employed both FFQ and 3-day 24-hour dietary recalls for dietary assessment**^[9]^**.
2) Monitoring period: Dietary recalls were conducted during the same time as fecal sample collection, ensuring temporal alignment between dietary exposure and microbiome/metabolome measurements.
3) Verification of dietary homogeneity: As presented in **Supplementary Table 2**, we found no statistically significant differences in daily per capita intake of energy, protein, carbohydrate, fat, dietary fiber, fat-soluble vitamins (A, E), water-soluble vitamins (B1, B2, B3, C, folic acid), and minerals (potassium, sodium, calcium, phosphorus) between HD patients and their cohabiting HC pairs (all *p* > 0.05). These results confirm that our household matching strategy successfully achieved comparable dietary intake between groups, effectively minimizing diet as a confounder.

We acknowledge the reviewer’ s important point that absolute dietary identity between HD patients and their healthy family members is not attainable in real-world settings. Indeed, HD patients often face dietary restrictions (e.g., potassium, phosphorus limitation) that may lead to minor differences in specific food choices. Nevertheless, the household paired design represents the best achievable control for dietary and environmental confounding in observational human studies^[10]^. This approach is widely recognized in the literature: a study on restricted diet in healthy volunteers showed that even under strictly controlled identical dietary conditions, individuals’ gut microbiomes do not converge toward a uniform composition, confirming that diet is only one of many factors shaping the gut microbiota. Thus, while we have controlled for dietary intake to the greatest extent feasible through cohabitation matching, the residual differences are likely minimal and do not compromise the validity of our primary conclusions regarding HD-associated dysbiosis.

We have incorporated the above clarifications into the revised Materials and Methods section (subsections“Study design and patient populatio”, “Dietary intake survey and evaluation”, and “Statistical analyses”). The specific modifications are shown in the revised manuscript.

We hope these revisions adequately address the reviewers’ concerns.

(3) Improvement of methodological transparency and reproducibility

Correct grammatical errors in the description of the study design, supplement complete information (manufacturer, catalog number, and country of origin) for all reagents and instruments, and ensure that the full analytical workflow for 16S rRNA sequencing and metabolomics is described in sufficient detail to support independent replication of the experiment by other researchers, in compliance with the transparency requirements of PLOS ONE.

Response: We thank the reviewer for these important suggestions to improve the methodological transparency and reproducibility of our study. We have carefully revised the manuscript as follows:

(1) Grammatical errors: We have corrected the identified grammatical issues in the *“Study design and patient population”* section and refined the sentence structures. The revised text now reads clearly and professionally. **(Page 6, Line 105-114)**

(2) Complete reagent and instrument information: We have systematically supplemented the manufacturer, catalog number, and country of origin for all key reagents and instruments used in this study, including: **(Page 7, Line 149-150, 157-158, 159-185, 204-207, 211-214)**

a. Fecal Genome DNA Extraction Kit (BioTeke, AU46111-96, China)
b. Phusion High-Fidelity PCR Master Mix (Thermo Fisher, F531L, USA)
c. AMPure XT beads (Beckman Coulter, A63882, USA)
d. Illumina NovaSeq 6000 (Illumina, USA)
e. ACQUITY UPLC HSS T3 column (Waters, 186003539, UK)
f. Orbitrap Exploris 120 mass spectrometer (Thermo Fisher, BRE725536, Germany)
g. HPLC-grade methanol (Honeywell, 34860, USA)
h. Milli-Q water purification system (Merck, ZMQSV0T0, Germany)
i. Automated biochemistry analyzer (Mindray, BS-360S, China)

And others as detailed in the revised Materials and Methods.

(3) Detailed analytical workflows: We have substantially expanded the description of both 16S rRNA sequencing and metabolomics workflows to ensure independent reproducibility. Specifically:

a. For 16S sequencing: “16S rRNA gene amplification and sequencing” and “Bioinformatics analysis of 16S rRNA data” within the Materials and Methods section. This includes: PCR reaction conditions (volumes, primer concentrations, thermal cycler parameters), quality filtering thresholds (Q20 sliding window, length >100 bp, <5% Ns), chimera removal (VSEARCH uchime_denovo), ASV inference (DADA2 parameters), taxonomic assignment criteria (SILVA confidence≥0.7; NT-16S identity≥90%, coverage≥80%, E-value≤1e-5), and all software versions.**(Page 8, Line159-170, 171-185)**
b. For metabolomics: “Metabolome sample preparation”, “LC-MS analysis”, and “Metabolomics data processing and statistical analysis” within the Materials and Methods section. This includes: detailed extraction protocol, LC gradient program (complete time points and solvent percentages), MS acquisition parameters (resolution, AGC targets, injection times, DDA settings), data processing steps (XCMS parameters, annotation databases HMDB and KEGG, CAMERA and metaX packages), and statistical criteria for differential metabolite selection (paired t-test *p* < 0.05, |FC| ≥ 1.2, VIP ≥ 1). **(Page 9-10, Line 196-203, 204-220, 221-244)**

(4) Software versions: All software and R packages are now reported with specific version numbers, including Cutadapt (v1.9), FLASH (v1.2.8), fqtrim (v0.94), VSEARCH (v2.3.4), DADA2 (v1.18.0), QIIME2 (v2021.11), R (v4.3.3), phyloseq (v1.38.0), ade4 (v1.7-22), metaX (v1.6.0), ropls (v1.30.0), CAMERA (v1.46.0), pROC (v1.15.0), clusterProfiler (v4.8.0), and SPSS (v26.0). **(Page 11, Line 262-266)**

All modifications have been highlighted in the revised manuscript. We believe these changes now fully meet the transparency and reproducibility standards required by PLOS ONE. We thank the reviewer again for their constructive feedback.

**Comment 3:**

Revision of Academic Expression and Logical Consistency Across All Manuscript Sections

(1) Introduction Section

Resolve the logical contradiction between the introduction and study results: The current statement in the introduction that HD patients "have a dramatic decrease in gut microbial diversity" is directly inconsistent with the study findings (significantly higher Chao1 index in the HD group, no difference in the Shannon index). The authors must revise this statement, systematically summarize inconsistent conclusions on gut microbial diversity in HD patients from published studies, and refine the study hypothesis, rather than presenting "reduced diversity" as an established conclusion.
Strengthen the clinical theoretical rationale for the study: As recommended by Reviewer 3, supplement published research evidence linking gut microbiome alterations in HD patients to adverse clinical outcomes (including mortality, cardiovascular events, malnutrition, and inflammation), to fully demonstrate the clinical importance of this study.

Correct non-standard academic expressions: Replace "quantity" with "number", rewrite the study objectives, remove academically weak phrases such as "attempted and new insight", and adopt the rigorous expression recommended by Reviewer 2 to clearly define the primary and secondary endpoints of the study.

Response: We thank the Editor and Reviewers for their careful reading of our Introduction and for raising important concerns regarding logical consistency, clinical rationale, and academic language. We have substantially revised the Introduction as follows.

(1) Resolution of the logical contradiction between the Introduction and study results

Reviewer’ s concern: The original Introduction stated that HD patients “*have a dramatic decrease in gut microbial diversity*” as an established fact, which directly contradicts our own findings (significantly higher Chao1 index and no difference in Shannon index in the HD group).

Our revision: We have removed the absolute statement. The revised Introduction now objectively summarizes the inconsistent findings in the literature: some studies report decreased diversity, whereas others have reported increased richness or no significant differences. We explicitly state that these discrepancies may arise from differences in study populations, dietary habits, living environments, and insufficient control of confounding factors. This revised presentation acknowledges the existing controversy and positions our study as a re-evaluation under rigorously controlled conditions (identical diet and living environment via household pairing).

(2) Strengthening of the clinical theoretical rationale

Reviewer’s concern (Reviewer #3)**:** The introduction would benefit from a stronger rationale for studying gut dysbiosis specifically in hemodialysis patients, emphasizing the clinical relevance (mortality, cardiovascular events, malnutrition, inflammation).

Our revision: We appreciate this point. The reviewer is right that the clinical importance of gut dysbiosis in HD patients needed to be spelled out more clearly. Therefore, we went back and searched the literature more carefully, and we found several high-quality studies that directly link gut microbiome alterations in HD patients to adverse outcomes.

Based on that, we revised one sentence in the Introduction to explicitly state that taxonomic and functional changes in the gut microbiome have been linked to multiple conditions, including inflammatory bowel disease (IBD)^[11-12]^, malnutrition**^[13,17]^**, cardiovascular events**^[14]^**, higher mortality risk**^[15-16]^**, and metabolic disorders**^[18]^**. For each of these associations, we cited recent, high quality studies that we identified through PubMed. We made sure the citations cover both the general IBD literature and, more importantly, the evidence specifically from CKD/hemodialysis populations regarding malnutrition, cardiovascular complications, and increased mortality.

We believe this revision makes the clinical relevance of our study much clearer and directly addresses the reviewer’s concern. The added sentence now reads:“Taxonomic and functional changes in the GIT microbiome have been linked to numerous illnesses, including inflammatory bowel disease (IBD), malnutrition, cardiovascular events, higher mortality risk , and metabolic disorders.”We thank the reviewer for pushing us to improve this part. (We have adjusted the citation numbers according to the updated reference list.) **(Page 4, Line 73-75)**

(3) Correction of non-standard academic expressions

Reviewer’s concern (Reviewer #2): Replace “quantity” with “number”; remove weak phrases such as “attempted” and “new insight”; clearly define primary and secondary endpoints. **(Page 4, Line 67)**

Our revision: We have made the following changes:

“*quantity*” has been replaced with “*number*” (now in the corrected epidemiological data sentence).

The final paragraph of the Introduction has been completely rewritten. We have removed the weak phrasing *“We attempted to discover novel therapeutic targets and new insights”*. Instead, we now clearly define the primary and secondary objectives as recommended: “*Therefore, the primary objective of this study was to evaluate differences between HD patients and HC individuals in gut microbial diversity, fecal metabolite profiles, and associated metabolic pathways using a 1:1 matched case-control design. The secondary objective was to perform integrated multi-omics analysis to examine correlations among identified microbial species, metabolites, and functional pathways, thereby generating hypotheses for future mechanistic and translational studies.”***(Page 5, Line 99-103)**

We believe the revised Introduction is now logically consistent with our results, clinically relevant, and written in proper academic style. We greatly appreciate the reviewers’ guidance, which has significantly improved the clarity and rigor of our manuscript.

(2) Results Section

Correct all group nomenclature errors: Uniformly replace “healthy families” with "healthy controls (HC)", revise "HC patients" to "HD patients", and ensure consistent group nomenclature throughout the manuscript to avoid reader confusion.

Conduct a comprehensive review of the accuracy of all data, statistical values, and *p*-values in the Results section, to ensure full consistency between the main text, figures, tables, and supplementary materials.

**Response:** We apologize for the careless mistakes in the Results section. We have now gone through the entire manuscript very carefully and corrected all group naming errors.

Specifically, we replaced *“healthy families”* with *“healthy controls (HC)”* **(Page 12, Line 278)** and changed the incorrect *“HC patients”* to *“HD patients”* **(Page 13, Line 309)**. We also checked every other place where group names appear, including figures and supplementary materials, to make sure “HD” and “HC” are used consistently throughout.

In addition, we re‑checked all statistical values and *p*‑values in the Results against the original analysis outputs and the supplementary tables. We found a couple of minor mismatches (e.g., age *p*‑value and BMI *p*‑value between the main text and Supplementary Table 1) and have corrected them so that the numbers are now fully consistent. We also verified that all figure and table citations in the Results point to the correct items.

We thank the reviewer for pointing these issues out. The manuscript is now much cleaner and more professional.

(3) Conclusion Section

Restructure the conclusion as recommended by Reviewer 2: Avoid restating detailed content from the Discussion section, concisely summarize 3-4 core findings of the study, highlight the methodological strengths and innovation of the work, provide clear implications for clinical practice, and ensure that all conclusions are strictly based on the study data without overinterpretation.

Response: Thank you for pointing out the issues with the Conclusion. I read through the entire Results section again, and I agree that the original conclusion was too wordy and repeated too many details that belong in the Discussion (for example, listing all four pathway names). Also, it did not highlight the methodological strength of our study.

We have rewritten the conclusion. Instead of a long paragraph, I now present the main findings as a short numbered list with four points. Each point corresponds to one key message: **(Page 22, Line 525-536)**

*We have substantially revised this section as follows: “*

*1.HD patients exhibited higher gut microbial richness (Chao1 index) but no significant difference in Shannon diversity compared with controls, with enrichment of Ruminococcus and depletion of Bifidobacterium.*

*2.A total of 497 fecal metabolites were significantly altered in HD patients, predominantly involving amino acid, vitamin, and lipid metabolism.*

*3.Integrated multi‑omics analysis revealed correlations between specific gut bacteria and metabolites, and identified four shared dysregulated functional pathways: Bile secretion, Caffeine metabolism, Gastric acid secretion, and Vitamin B6 metabolism.*

*4.The fecal metabolites 4‑pyridoxic acid, imidazoleacetic acid, and ascorbic acid showed high diagnostic accuracy and may serve as non‑invasive candidate biomarkers for HD”*

We also added one sentence at the beginning to highlight our design (household‑paired, same diet and living environment), because that is a key strength of this study. I believe this version is much clearer, more focused, and better aligned with what the journal expects. Thank you again for the helpful suggestion

**Comment 4:**

Comprehensive Elaboration of Innovation, Clinical Translation Value, and Study Limitations

(1) Clear elaboration of the study’s innovation

The authors must add a dedicated subsection in the Discussion to explicitly address the questions raised by Reviewer 4, including:

Whether the paired design under shared dietary and living conditions is the core innovation of the study, and its advantages over previously published studies;

The innovation of the multi-omics integration analysis in this study, especially its advancement to the field compared with previous single-omics studies, in terms of microbiome-metabolome interactions and shared differential pathways;

The advantages of the diagnostic biomarkers identified in this study over previously reported markers related to HD patients.

Response: Thank you for raising these important points. I have now added a dedicated subsection in the Discussion titled “*Innovations”* to address your questions directly.

(1) Regarding the paired design:

I agree that this is the core innovation. I have made it clear that most previous case‑control studies did not control for diet and living environment, which are major confounders. By matching each HD patient with a family member living in the same household, we reduced those confounders as much as possible. This design is not perfect but it is a real step forward compared to studies that simply compare HD patients to unrelated healthy volunteers.

(2) Regarding multi‑omics integration:

I have now explained that instead of doing only 16S sequencing or only serum metabolomics (which most previous studies did), we combined fecal microbiome and fecal metabolome data. This allowed us to see correlations between specific bacteria and metabolites, and to find four functional pathways that were altered in both data sets. As far as I know, this kind of cross‑omics convergence has not been reported before in HD patients.

(3) Regarding the diagnostic biomarkers:

I have highlighted that our candidate biomarkers (4‑pyridoxic acid, imidazoleacetic acid, ascorbic acid) are fecal markers. They are non‑invasive and easy to collect, which is a clear advantage over serum markers that require a blood draw. I also mentioned that they directly reflect gut microbial activity. Of course, external validation is still needed, but the practical advantages are worth pointing out.

I have kept the tone honest and not overclaimed anything. The new subsection is placed right before the *“Clinical translation feasibility”* paragraph, thereby maintaining a logical and natural flow. I believe this now fully addresses your concerns. **(Page 20, Line 480-491)**

Thank you again for pushing me to clarify these points.

(2) Analysis of the clinical translation feasibility of the biomarkers

Explicitly state that the markers identified in this study are fecal markers, and supplement the advantages and limitations of fecal markers compared with systemic (serum) markers in the clinical practice of HD.

As requested by Reviewer 4, detail the feasibility of applying these markers in routine HD clinical practice, including the accessibility of detection methods, cost, convenience of specimen collection, and the clinical value of their diagnostic performance.

Response: You asked me to clarify whether these are fecal or systemic markers, and to discuss the feasibility of using them in routine practice. I have added a new subsection *“cinical translation feasibility”* in the Discussion to address this. **(Page 20-21, Line 492-499)**

First, I clearly stated that all of our candidate biomarkers are from fecal samples. Then I compared them to serum markers: the main advantages are non-invasive collection, and patients can do it at home. The fact directly reflects gut microbial metabolism, rather than being diluted or modified by the host after absorption.

But I also did not hide the limitations. Fecal samples can vary depending on stool consistency and transit time, and storage conditions matter. LC-MS is still expensive and not available in every hospital lab. So, I suggested that a more realistic path for clinical use would be to develop simpler targeted tests for the top metabolites, such as ELISA or point-of-care kits. This still requires external validation.

Despite these hurdles, the diagnostic performance of the combined panel (AUC 0.935) is promising enough to consider these markers as a noninvasive screening tool. I also mentioned that cost effectiveness studies are needed before routine adoption.

I think this gives a balanced and honest view of where we stand. Thank you for pushing me to consider the practical application more carefully.

(3) Supplementation of potential intervention strategies

Add a dedicated paragraph in the Discussion section to discuss potential intervention strategies for gut dysbiosis in HD patients (including probiotics, vitamin supplementation, and dietary modulation) based on the differential bacteria, metabolites, and pathways identified in this study, to enhance the clinical guidance value of the work.

Response: Thanks for this suggestion. I agree that discussing possible interventions makes the findings more useful for clinicians. Thus, I added a short sentence in the Discussion to briefly mention vitamin B6/B3 supplementation, dietary fiber, and probiotics as directions worth exploring, based on our results. I kept it realistic and made clear that these still need to be tested specifically in HD patients. We have substantially revised this section as follows: *“Our results suggest that vitamin B6, B3, C, and biotin supplementation might help restore metabolic balance, while increasing dietary fiber could support Bifidobacterium and short‑chain fatty acid(SCFA) production. We could also consider using possible probiotics, such as Bifidobacterium. These strategies aim to counter the observed dysbiosis and metabolic shifts, but need validation specifically in HD patients”***.(Page 21, Line 500-504)**

(4) Comprehensive and objective statement of study limitations

As required by Reviewer 4, the authors must comprehensively supplement the limitations of this study in the dedicated limitations subsection of the Discussion, including but not limited to: The cross-sectional study design cannot establish a causal relationship between gut microbiome alterations and HD status;

The use of 16S rRNA sequencing rather than shotgun metagenomic sequencing limits the species-level taxonomic resolution and functional annotation capability;

The lack of serum metabolomics data prevents the association of fecal microbiome and metabolome alterations with the host’s systemic metabolic status;

The geographical limitation of the multi-center cohort (all centers located in Hebei Province, China) restricts the generalizability of the study findings;

The identified diagnostic biomarkers lack validation in an external cohort;

Other limitations, including the inability to fully exclude the long-term effects of prior antibiotic use, and single-timepoint sampling that cannot capture the dynamic changes of the gut microbiome and metabolome.

Response: Thanks for listing the specific limitations we need to include. I have expanded the limitations paragraph in the Discussion to cover all the points you raised:

cross‑sectional design (no causal inference);

16S rRNA instead of shotgun sequencing;

lack of serum metabolomics;

geographic limitation to Hebei Province;

no external validation for the biomarkers;

I kept it concise and honest, without trying to hide any of these weaknesses.

We have substantially revised this section as follows: *“There are various limitations to our investigation. First, due to the observational design, our findings cannot indicate causal links. Second, only baseline fecal samples were taken. However, since microbial diversity may decrease with time in HD patients, a higher baseline microbial diversity could bias the study results in favor of the null hypothesis. Third, the long-term effects of past antibiotic use on gut microbiota cannot be fully excluded. Fourth, limited by the depth and scope of the study, we have not yet conducted in-depth research on the underlying mechanisms that affect the disease process and prognosis of patients. We also acknowledge several technical limitations. Using 16S rRNA sequencing instead of shotgun metagenomics limited species level resolution and functional prediction. The absence of serum metabolomics means we cannot directly connect fecal changes to the host's systemic metabolic status. Since all participants came from Hebei Province, our findings may not generalize to other regions”*.**(Page 21, Line 510-520)**

**Comment 5:**

Supplementary Requirements for Figures, Tables, and Data Visualization

(1) Based on the results of Spearman correlation analysis, add a correlation network diagram illustrating the interactions between differential gut bacterial genera and differential metabolites, with clear annotation of correlation coefficients and statistical significance.

Response: We already have Supplementary Fig. 7 (Mantel test network heatmap) showing interactions between differential genera and metabolites, with correlation coefficients and *p*-values. We have now added a correlation network diagram as a new supplementary figure (Supplementary Fig. 7). In this figure, green triangles represent differential bacterial genera, purple circles represent differential metabolites. Lines between them indicate significant *Spearman* correlations (*p* < 0.05): solid lines for positive correlations, dashed lines for negative correlations. The diagram clearly shows the main interactions, for example between *Ruminococcus* and several lipid‑related metabolites. We believe this visualization helps readers grasp the multi‑omics connections more intuitively. I made sure the annotation is clear. **(Page 32, Line 780-783)**

(2) Add a heatmap of significantly altered metabolites (core differential metabolites) to visually demonstrate the differences in metabolic profiles between the HD and HC groups.

Response: Supplementary Fig. 4 already shows this. It displays the top 20 differential metabolites with red/blue indicating up/down regulation. However, you were right that the original heatmap Supplementary Fig. 4 was not very clear. We went back and made a new one. This time we selected only the top 10 most significant differential metabolites rather than showing too much, which makes the differences between HD and HC clearer. The new heatmap is now included as Supplementary Fig. 4 (replacing the old one), and we updated the in-text citation accordingly. Thanks for pushing us to improve this figure. **(Page 31, Line 766-770)**

**S4 Fig. Metabolic patterns in hemodialysis patients and healthy controls.** Fecal metabolite profiles in hemodialysis patients and healthy controls were shown as heatmaps. Each row represented data for a differentially expressed metabolite and each column represented an individual. Different colors corresponded to the different relative abundance of metabolites. Red and blue colors represented increased and decreased levels of metabolites, respectively.

(3) Conduct a comprehensive review of all existing figures and tables: correct spelling errors (e.g., revise "hemodiaylsis" to "hemodialysis"), ensure complete legends and abbreviation annotations, standardized labeling of statistical values, and explicit in-text citations for all figures and tables. Ensure that all supplementary figures and tables have complete annotations and fully correspond to their citations in the main text.

Response: We have carefully checked every figure legend to ensure that each is complete and clearly presents the results. I corrected the spelling error "hemodiaylsis" to "hemodialysis" in all figure captions (S6, S7, S8, S11). I also checked all in‑text citations to make sure every figure and table is cited where it should be, and added missing annotations for abbreviations in the captions.

All changes are minor but should make the presentation cleaner.

**Comment 6:**

Standardization of Ethical Approval, Funding, and Data Availability Statements

(1) Ethics Statement: As requested by Reviewer 3, supplement complete details of ethical approval, including the full name of the ethics committee, approval number, type of consent obtained from participants (written informed consent), and a clear statement on whether a waiver of informed consent was granted (accompanied by the ethics committee’s approval document if applicable).

Response: Thank you for checking these administrative details.

I have added the full ethics committee name, approval number, and a clear statement that written informed consent was obtained from all participants and no waiver was granted. This is now in the Methods section. **(Page 6, Line 110-114)**

(2) Data Availability Statement: Revise the statement to comply with the open data policy of PLOS ONE. The authors must deposit the raw 16S rRNA sequencing data in a public repository (e.g., NCBI Sequence Read Archive (SRA), ENA, DDBJ), and the raw metabolomics data in a specialized metabolomics database (e.g., MetaboLights, Metabolomics Workbench). The dataset accession numbers must be provided in the revised manuscript to ensure unrestricted public access to all underlying data.

Response: Thank you for the reminder. I understand the journal requires raw data to be publicly accessible. After checking available options, I have deposited the raw 16S rRNA and metabolomics data in Science Data Bank (https://www.scidb.cn), which is a public repository fully compliant with PLOS ONE's open data policy. The DOI is [10.57760/sciencedb.34128]. I have updated the *“Data Availability Statement”* in the manuscript accordingly. I apologize for the inconvenience and hope this meets the policy.

**(Page 23, Line 547-550)**

(3) Competing Interests Statement: Ensure that the competing interests statement is consistent with the content in the submission system, and explicitly declare that no authors have relevant competing interests to disclose.

Response: We corrected the statement to match the journal’s format: *“The authors have declared that no competing interests exist”.* This is now consistent with the submission system. **(Page 23, Line 555)**

All changes have been highlighted in the revised manuscript.

In conclusion, we extend our sincere gratitude to the editor and all reviewers for your insightful comments and constructive suggestions. After carefully revising our manuscript, we believe that it has been significantly improved. If you have any further questions or concerns, please do not hesitate to point them out, we are fully committed to making additional revisions as needed. Once again, we appreciate your time and effort in reviewing our work.

**Reference**

[1] Chen XM. National Data Report on Dialysis in China 2024. Presented at: 19th Conference on Critical Kidney Disease and Blood Purification, Chinese Society of Nephrology; July 2-5, 2025; Tianjin, China.
[2] Zhang L, Zuo L, Wang Y, Yu F, Yang C, Zhao MH, et al. China Kidney Disease Network (CK‑NET) 2017–2018 Annual Data Report. Kidney Int Suppl. 2025;14(1):e1‑e133. doi:10.1016/ j.kisu.2024.12.001
[3] Jiao Y, Wolf M, Croft KR, Carioni P, Soni M, Stuard S, et al. Kidney failure etiology and modality worldwide: Apollo Dial DB: FR-PO393. J Am Soc Nephrol. 2024;35(10S). doi: 10.1681/ASN.20244yvbgswh.
[4] Okpechi IG, Levin A, Tungsanga S, Arruebo S, Caskey FJ, Chukwuonye II, et al. Progress of nations in the organisation of, and structures for, kidney care delivery between 2019 and 2023: cross sectional survey in 148 countries. BMJ. 2024 Oct 14;387:e079937. doi: 10.1136/bmj-2024-079937.
[5] Rothschild D, Weissbrod O, Barkan E, et al. Environment dominates over host genetics in shaping human gut microbiota. Nature. 2018;555(7695):210-215. doi:10.1038/nature25973
[6] The iMSMS Consortium, Zhou X, et al. Household paired design reduces variance and increases power in multi-city gut microbiome study in multiple sclerosis. Mult Scler. Published online June 26, 2020. doi:10.1177/1352458520924594
[7] Li C, Guo X, He Y, Wang J, Hao J, Liu X. Cohabiting with ulcerative colitis patients decreases differences of gut microbiome between healthy individuals and the patients. Ann Med. 2024;56(1):2337712. doi:10.1080/07853890.2024.2337712
[8] Mattiello F, Verbist B, Faust K, et al. A web application for sample size and power calculation in case-control microbiome studies. Bioinformatics. 2016;32(13):2038-2040. doi:10.1093/bioinformatics/btw099
[9] Verger, Eric O, Armstrong, Patrice, Nielsen, et al. Dietary Assessment in the MetaCardis Study: Development and Relative Validity of an Online Food Frequency Questionnaire. Journal of the Academy of Nutrition and Dietetics, S2212267216313351–. doi:10.1016/j.jand.2016.10.030
[10] Xu YS, Wang YH, Liu Y, et al. Alteration of the faecal microbiota composition in patients with constipation: evidence of American Gut Project. Benef Microbes. 2022;13(6):427-436. doi:10.3920/BM2022.0009
[11] Lloyd-Price J, Arze C, Ananthakrishnan AN, et al. Multi-omics of the gut microbial ecosystem in inflammatory bowel diseases. Nature. 2019 May;569(7758):655-662. doi: 10.1038/s41586-019-1237-9.
[12] Florio M, Crudele L, Sallustio F, et al. Disentangling the nutrition-microbiota liaison in inflammatory bowel disease. Mol Aspects Med. 2025 Apr;102:101349. doi: 10.1016/j.mam.2025.101349.
[13] Du J, Zhao X, Ding X, Han Q, Duan Y, Ren Q, et al. The Role of the Gut Microbiota in Complications among Hemodialysis Patients. Microorganisms. 2024 Sep 12;12(9):1878. doi: 10.3390/microorganisms12091878.
[14] Sabatino A, Regolisti G, Cosola C, Gesualdo L, Fiaccadori E. Intestinal Microbiota in Type 2 Diabetes and Chronic Kidney Disease. Curr Diab Rep. 2017 Mar;17(3):16. doi: 10.1007/s11892-017-0841-z. PMID: 28271466.
[15] Lin TY, Wu PH, Lin YT, Hung SC. Gut dysbiosis and mortality in hemodialysis patients. NPJ Biofilms Microbiomes. 2021 Mar 3;7(1):20. doi: 10.1038/s41522-021-00191-x.
[16] Martín-Del-Campo F, Avesani CM, Stenvinkel P, et al. Gut microbiota disturbances and protein-energy wasting in chronic kidney disease: a narrative review. J Nephrol. 2023 Apr;36(3):873-883. doi: 10.1007/s40620-022-01560-1.
[17] DAS B, Nair GB. Homeostasis and dysbiosis of the gut microbiome in health and disease. J Biosci. 2019 Oct;44(5):117. doi: 10.1007/s12038-019-9926-y.
[18] Al Awadhi S, Myint L, Guallar E, et al. A Metabolomics Approach to Identify Metabolites Associated With Mortality in Patients Receiving Maintenance Hemodialysis. Kidney Int Rep. 2024 Jun 29;9(9):2718-2726. doi: 10.1016/j.ekir.2024.06.039.
