# Supplementary material for: Integrated multi-omics reveals dysbiosis in hemodialysis patients: A multi-center study
Source: PLoS One. 2026 Aug 13;21(8):e0355698. doi: 10.1371/journal.pone.0355698 (PMC13472434; doi:10.1371/journal.pone.0355698)
Supplement: Peer review history

# Peer review: Decision Letter 0

**DOI:** 10.1371/journal.pone.0355698.r001

**Authors:** Hongtao Bi

2 Mar 2026

Dear Dr. Di,

Thank you for submitting your manuscript to PLOS ONE. After careful consideration, we feel that it has merit but does not fully meet PLOS ONE’s publication criteria as it currently stands. Therefore, we invite you to submit a revised version of the manuscript that addresses the points raised during the review process.

Please submit your revised manuscript by Apr 16 2026 11:59PM. If you will need more time than this to complete your revisions, please reply to this message or contact the journal office at plosone@plos.org. When you're ready to submit your revision, log on to https://www.editorialmanager.com/pone/ and select the 'Submissions Needing Revision' folder to locate your manuscript file.

- A letter that responds to each point raised by the academic editor and reviewer(s). You should upload this letter as a separate file labeled 'Response to Reviewers'.
- A marked-up copy of your manuscript that highlights changes made to the original version. You should upload this as a separate file labeled 'Revised Manuscript with Track Changes'.
- An unmarked version of your revised paper without tracked changes. You should upload this as a separate file labeled 'Manuscript'.

If you would like to make changes to your financial disclosure, please include your updated statement in your cover letter. Guidelines for resubmitting your figure files are available below the reviewer comments at the end of this letter.

If applicable, we recommend that you deposit your laboratory protocols in protocols.io to enhance the reproducibility of your results. Protocols.io assigns your protocol its own identifier (DOI) so that it can be cited independently in the future. For instructions see: https://journals.plos.org/plosone/s/submission-guidelines#loc-laboratory-protocols. Additionally, PLOS ONE offers an option for publishing peer-reviewed Lab Protocol articles, which describe protocols hosted on protocols.io. Read more information on sharing protocols at https://plos.org/protocols?utm_medium=editorial-email&utm_source=authorletters&utm_campaign=protocols.

We look forward to receiving your revised manuscript.

Kind regards,

Hongtao Bi, Ph.D.

Academic Editor

PLOS One

**Journal Requirements:**

1. When submitting your revision, we need you to address these additional requirements.

Please ensure that your manuscript meets PLOS ONE's style requirements, including those for file naming. The PLOS ONE style templates can be found at

https://journals.plos.org/plosone/s/file?id=wjVg/PLOSOne_formatting_sample_main_body.pdf and

https://journals.plos.org/plosone/s/file?id=ba62/PLOSOne_formatting_sample_title_authors_affiliations.pdf

2. Please note that PLOS One has specific guidelines on code sharing for submissions in which author-generated code underpins the findings in the manuscript. In these cases, we expect all author-generated code to be made available without restrictions upon publication of the work. Please review our guidelines at https://journals.plos.org/plosone/s/materials-and-software-sharing#loc-sharing-code and ensure that your code is shared in a way that follows best practice and facilitates reproducibility and reuse.

3. We note that the grant information you provided in the ‘Funding Information’ and ‘Financial Disclosure’ sections do not match.

When you resubmit, please ensure that you provide the correct grant numbers for the awards you received for your study in the ‘Funding Information’ section.

4. Thank you for stating in your Funding Statement:

This work was supported by Hebei Provincial Health Commission and Department of Finance of Hebei Province (2024 Government-Sponsored Outstanding Medical Talent Program, No.ZF2024069)

Please provide an amended statement that declares *all* the funding or sources of support (whether external or internal to your organization) received during this study, as detailed online in our guide for authors at http://journals.plos.org/plosone/s/submit-now.  Please also include the statement “There was no additional external funding received for this study.” in your updated Funding Statement.

Please include your amended Funding Statement within your cover letter. We will change the online submission form on your behalf.

5. Thank you for stating the following financial disclosure:

This work was supported by Hebei Provincial Health Commission and Department of Finance of Hebei Province (2024 Government-Sponsored Outstanding Medical Talent Program, No.ZF2024069)

Please state what role the funders took in the study.  If the funders had no role, please state: "The funders had no role in study design, data collection and analysis, decision to publish, or preparation of the manuscript."

If this statement is not correct you must amend it as needed.

Please include this amended Role of Funder statement in your cover letter; we will change the online submission form on your behalf.

6. Thank you for stating the following in your Competing Interests section:

I have read the journal's policy and the authors of this manuscript have the following competing interests: [The authors have declared that no competing interests exist]

Please complete your Competing Interests on the online submission form to state any Competing Interests. If you have no competing interests, please state "The authors have declared that no competing interests exist.", as detailed online in our guide for authors at http://journals.plos.org/plosone/s/submit-now

This information should be included in your cover letter; we will change the online submission form on your behalf.

7. In the online submission form, you indicated that “The data underlying this article are available in the article and in its online supplementary material. Most of the data has been shown in the form of figures and tables in the article. The anonymized microbiome and metabolome datasets are available to other researchers upon request.”

All PLOS journals now require all data underlying the findings described in their manuscript to be freely available to other researchers, either a. In a public repository, b. Within the manuscript itself, or c. Uploaded as supplementary information.

This policy applies to all data except where public deposition would breach compliance with the protocol approved by your research ethics board. If your data cannot be made publicly available for ethical or legal reasons (e.g., public availability would compromise patient privacy), please explain your reasons on resubmission and your exemption request will be escalated for approval.

8. Your ethics statement should only appear in the Methods section of your manuscript. If your ethics statement is written in any section besides the Methods, please move it to the Methods section and delete it from any other section. Please ensure that your ethics statement is included in your manuscript, as the ethics statement entered into the online submission form will not be published alongside your manuscript.

9. Please upload a new copy of Figures 1 to 4, S4, S6, S7 and S8, as the detail is not clear. Please follow the link for more information:  https://journals.plos.org/plosone/s/figures

10. Please include captions for your Supporting Information files at the end of your manuscript, and update any in-text citations to match accordingly. Please see our Supporting Information guidelines for more information: http://journals.plos.org/plosone/s/supporting-information.

11. If the reviewer comments include a recommendation to cite specific previously published works, please review and evaluate these publications to determine whether they are relevant and should be cited. There is no requirement to cite these works unless the editor has indicated otherwise. 

**Additional Editor Comments:**

This manuscript employs an integrated multi-omics approach combining 16S rRNA gene sequencing and untargeted fecal metabolomics to conduct a 1:1 matched case-control study investigating the characteristics of gut dysbiosis in hemodialysis (HD) patients, with HD patients and cohabiting healthy controls (HC) sharing identical dietary and living conditions enrolled as the study subjects. The clinical question addressed by this study is of critical importance: the crosstalk between the gut microbiome and metabolome is closely associated with adverse clinical outcomes in HD patients, and the paired design adopted in this study effectively controls for diet, the most prominent confounding factor in gut microbiome research. The study has a relatively sufficient sample size (96 HD-HC matched pairs) and a rigorous multi-omics analytical workflow.

A total of 4 peer reviewers were invited to evaluate this manuscript during the review process. Reviewer 1 highly recognized this study, stating that the work has a rigorous design, reliable multi-omics methodology, and promising findings regarding the identified biomarkers, and recommended direct acceptance without revisions. However, Reviewers 2, 3, and 4 all raised multiple critical concerns, covering fundamental errors in epidemiological data and calculation formulas, inconsistent descriptions of the study design, insufficient methodological details, logical inconsistency between the introduction and study results, inadequate elaboration of innovation and clinical translation value, missing data visualization figures, and incomplete disclosure of study limitations, among other aspects.

As the Academic Editor handling this manuscript, I have comprehensively evaluated the full text of the manuscript and all reviewer comments. I confirm that this study has scientific value and clinical relevance, but there are major flaws in academic rigor, methodological accuracy, and content completeness, which must be fully addressed through a major revision.

1. Correction of the Accuracy of Core Data and Epidemiological Statements

(1) Correction of prevalence data for hemodialysis patients

The statement in the manuscript that "the number of HD patients in China had reached 8.44 million by the end of December 2022" has a fundamental deviation from authoritative epidemiological data. As pointed out by Reviewer 3, this figure corresponds to the total prevalence of chronic kidney disease (CKD) in China, not the number of patients receiving maintenance hemodialysis. The authors must recheck the data from authoritative sources, including the China Kidney Disease Network (CK-NET) Annual Report and the National Medical Quality Report on Kidney Diseases issued by the National Health Commission of the People’s Republic of China, revise this figure, clearly distinguish between the total CKD population and maintenance hemodialysis patient population, and provide valid peer-reviewed references to support the revised data.

(2) Correction of the overgeneralized statement on renal replacement therapy modalities

The claim in the manuscript that "hemodialysis accounts for nearly 90% of patients receiving renal replacement therapy in most countries" is inaccurate and overly generalized, as the distribution of dialysis modalities varies significantly across countries, regions, and healthcare systems. The authors must revise this statement, provide region-specific data supported by appropriate references, or add rigorous qualifiers to avoid overgeneralization.

(3) Correction of errors in key calculation and statistical formulas

The reversed BMI calculation formula on Line 139 of the manuscript is a serious and unacceptable error in epidemiological research. The authors must immediately correct the formula to: Body Mass Index (BMI) = weight (kg) / height² (m²). In addition, as noted by Reviewer 2, the authors must conduct a comprehensive review of all statistical formulas, descriptions of fold-change (FC) thresholds, and screening criteria for differential features in the manuscript, correct all errors, and ensure 100% consistency between the methodological description and the actual analytical workflow.

2. Resolution of Core Issues in Study Design and Methodology

(1) Elimination of contradictory descriptions of the study type

The manuscript simultaneously describes the study as both a "prospectively recruited matched case-control study" and a "retrospective study", which constitutes a fundamental contradiction that directly affects the level of evidence and ethical compliance of the study. The authors must explicitly define the study type:

If it is a retrospective study: Supplement full details of the retrospective design, the time frame of data collection, and the approval status of the waiver of informed consent by the ethics committee (if applicable).

If it is a prospective case-control study: Delete all references to "retrospective study" in the manuscript, supplement full details of the prospective recruitment process and the implementation of inclusion and exclusion criteria, and ensure completely consistent description of the study type throughout the manuscript.

(2) Supplementation of details on the matching strategy and confounding factor control

As pointed out by Reviewer 3, the description of the 1:1 matching strategy is overly vague. The authors must supplement the following content in the Materials and Methods section:

Additional matching variables beyond age (±2 years) and sex, as well as the rationale for selecting the paired design;

The rationale for selecting paired statistical tests (paired t-test / Wilcoxon signed-rank test), and the results of statistical power verification for the paired design;

The specific method of dietary assessment (e.g., 3-day 24-hour dietary recall method, food frequency questionnaire), the time frame of dietary monitoring, and the statistical results verifying dietary homogeneity between cohabiting HD-HC pairs, to validate the core advantage of the study design and address the reviewers’ concerns about the effectiveness of dietary control.

(3) Improvement of methodological transparency and reproducibility

Correct grammatical errors in the description of the study design, supplement complete information (manufacturer, catalog number, and country of origin) for all reagents and instruments, and ensure that the full analytical workflow for 16S rRNA sequencing and metabolomics is described in sufficient detail to support independent replication of the experiment by other researchers, in compliance with the transparency requirements of PLOS ONE.

3. Revision of Academic Expression and Logical Consistency Across All Manuscript Sections

(1) Introduction Section

Resolve the logical contradiction between the introduction and study results: The current statement in the introduction that HD patients "have a dramatic decrease in gut microbial diversity" is directly inconsistent with the study findings (significantly higher Chao1 index in the HD group, no difference in the Shannon index). The authors must revise this statement, systematically summarize inconsistent conclusions on gut microbial diversity in HD patients from published studies, and refine the study hypothesis, rather than presenting "reduced diversity" as an established conclusion.

Strengthen the clinical theoretical rationale for the study: As recommended by Reviewer 3, supplement published research evidence linking gut microbiome alterations in HD patients to adverse clinical outcomes (including mortality, cardiovascular events, malnutrition, and inflammation), to fully demonstrate the clinical importance of this study.

Correct non-standard academic expressions: Replace "quantity" with "number", rewrite the study objectives, remove academically weak phrases such as "attempted and new insight", and adopt the rigorous expression recommended by Reviewer 2 to clearly define the primary and secondary endpoints of the study.

(2) Results Section

Correct all group nomenclature errors: Uniformly replace "healthy families" with "healthy controls (HC)", revise "HC patients" to "HD patients", and ensure consistent group nomenclature throughout the manuscript to avoid reader confusion.

Conduct a comprehensive review of the accuracy of all data, statistical values, and p-values in the Results section, to ensure full consistency between the main text, figures, tables, and supplementary materials.

(3) Conclusion Section

Restructure the conclusion as recommended by Reviewer 2: Avoid restating detailed content from the Discussion section, concisely summarize 3-4 core findings of the study, highlight the methodological strengths and innovation of the work, provide clear implications for clinical practice, and ensure that all conclusions are strictly based on the study data without overinterpretation.

4. Comprehensive Elaboration of Innovation, Clinical Translation Value, and Study Limitations

(1) Clear elaboration of the study’s innovation

The authors must add a dedicated subsection in the Discussion to explicitly address the questions raised by Reviewer 4, including:

Whether the paired design under shared dietary and living conditions is the core innovation of the study, and its advantages over previously published studies;

The innovation of the multi-omics integration analysis in this study, especially its advancement to the field compared with previous single-omics studies, in terms of microbiome-metabolome interactions and shared differential pathways;

The advantages of the diagnostic biomarkers identified in this study over previously reported markers related to HD patients.

(2) Analysis of the clinical translation feasibility of the biomarkers

Explicitly state that the markers identified in this study are fecal markers, and supplement the advantages and limitations of fecal markers compared with systemic (serum) markers in the clinical practice of HD.

As requested by Reviewer 4, detail the feasibility of applying these markers in routine HD clinical practice, including the accessibility of detection methods, cost, convenience of specimen collection, and the clinical value of their diagnostic performance.

(3) Supplementation of potential intervention strategies

Add a dedicated paragraph in the Discussion section to discuss potential intervention strategies for gut dysbiosis in HD patients (including probiotics, vitamin supplementation, and dietary modulation) based on the differential bacteria, metabolites, and pathways identified in this study, to enhance the clinical guidance value of the work.

(4) Comprehensive and objective statement of study limitations

As required by Reviewer 4, the authors must comprehensively supplement the limitations of this study in the dedicated limitations subsection of the Discussion, including but not limited to:

The cross-sectional study design cannot establish a causal relationship between gut microbiome alterations and HD status;

The use of 16S rRNA sequencing rather than shotgun metagenomic sequencing limits the species-level taxonomic resolution and functional annotation capability;

The lack of serum metabolomics data prevents the association of fecal microbiome and metabolome alterations with the host’s systemic metabolic status;

The geographical limitation of the multi-center cohort (all centers located in Hebei Province, China) restricts the generalizability of the study findings;

The identified diagnostic biomarkers lack validation in an external cohort;

Other limitations, including the inability to fully exclude the long-term effects of prior antibiotic use, and single-timepoint sampling that cannot capture the dynamic changes of the gut microbiome and metabolome.

5. Supplementary Requirements for Figures, Tables, and Data Visualization

(1) Based on the results of Spearman correlation analysis, add a correlation network diagram illustrating the interactions between differential gut bacterial genera and differential metabolites, with clear annotation of correlation coefficients and statistical significance.

(2) Add a heatmap of significantly altered metabolites (core differential metabolites) to visually demonstrate the differences in metabolic profiles between the HD and HC groups.

(3) Conduct a comprehensive review of all existing figures and tables: correct spelling errors (e.g., revise "hemodiaylsis" to "hemodialysis"), ensure complete legends and abbreviation annotations, standardized labeling of statistical values, and explicit in-text citations for all figures and tables. Ensure that all supplementary figures and tables have complete annotations and fully correspond to their citations in the main text.

6. Standardization of Ethical Approval, Funding, and Data Availability Statements

(1) Ethics Statement: As requested by Reviewer 3, supplement complete details of ethical approval, including the full name of the ethics committee, approval number, type of consent obtained from participants (written informed consent), and a clear statement on whether a waiver of informed consent was granted (accompanied by the ethics committee’s approval document if applicable).

(2) Data Availability Statement: Revise the statement to comply with the open data policy of PLOS ONE. The authors must deposit the raw 16S rRNA sequencing data in a public repository (e.g., NCBI Sequence Read Archive (SRA), ENA, DDBJ), and the raw metabolomics data in a specialized metabolomics database (e.g., MetaboLights, Metabolomics Workbench). The dataset accession numbers must be provided in the revised manuscript to ensure unrestricted public access to all underlying data.

(3) Competing Interests Statement: Ensure that the competing interests statement is consistent with the content in the submission system, and explicitly declare that no authors have relevant competing interests to disclose.

[Note: HTML markup is below. Please do not edit.]

Reviewers' comments:

**Comments to the Author**

1. Is the manuscript technically sound, and do the data support the conclusions?

Reviewer #1: Yes

Reviewer #2: Yes

Reviewer #3: Yes

Reviewer #4: Partly

**********

2. Has the statistical analysis been performed appropriately and rigorously?

Reviewer #1: Yes

Reviewer #2: No

Reviewer #3: Yes

Reviewer #4: I Don't Know

**********

3. Have the authors made all data underlying the findings in their manuscript fully available?

The PLOS Data policy

Reviewer #1: Yes

Reviewer #2: Yes

Reviewer #3: Yes

Reviewer #4: Yes

**********

4. Is the manuscript presented in an intelligible fashion and written in standard English?

Reviewer #1: Yes

Reviewer #2: Yes

Reviewer #3: Yes

Reviewer #4: No

**********

**Reviewer #1:**  This is an impressive study. The use of multi-omics (combining microbiome and metabolome data) gives a very detailed view of the health of hemodialysis patients.

I particularly appreciate the study design—matching patients with healthy controls under similar dietary conditions is excellent and makes your results much more reliable. The identification of specific biomarkers with high accuracy (AUC > 0.8) is a very promising finding.

I have no changes to request. This is a high-quality contribution to the field.

**Reviewer #2:**  Integrated Multi-Omics Reveals Dysbiosis in Hemodialysis Patients: A Multi-Center Study

INTRODUCTION

STRENGTHS

1. It has a clear and appropriate opening which is coherent and well structured.

2. On line 1033-104, the research gap is clearly stated.

WEAKNESSES

1. On line 75, The wording “quantity” is not appropriate academic language. Kindly replace it with “NUMBER”.

2. On line 84-86, The introduction presents decreased diversity as established, but the results later differ. The introduction does not acknowledge inconsistency.

I recommend you change “have a dramatic decrease in gut microbial diversity” to “Have reported alterations in gut microbial activity”.

3. On line 113-115, there is a vague statement that needs to be reconstructed

“Attempted and new insight” are weak academically. Kindly rephrase it like:

“We aimed to evaluate differences in gut microbial diversity, fecal metabolites, and metabolic pathways between HD patients and healthy controls, and to examine correlations among microbial species, metabolites, and functional pathways.”

MATERIALS AND METHODS

STRENGTH

1. The study design is clearly described, and variables are clearly matched and stated.

2. On line 122-124, confounders are well controlled.

3. The method is very transparent.

WEAKNESS

1. On line 117-118, there is a grammatical error “We conducted a 1:1 matched case-control study that hemodialysis (HD) patients were paired with healthy controls (HC)”

I recommend you construct it as: “We conducted a 1:1 matched case-control study in which hemodialysis (HD) patients were paired with healthy controls under….

2. On line 130, it was stated “For this retrospective study” But earlier description stated an active recruitment and matching. Kindly clarify and be consistent in wording. I recommend “IF Retrospective rephrase earlier sentence to match with the description. IF NOT Kindly remove the word “Retrospective”.

3. On Line 139, “The body mass index, or BMI, was calculated by dividing the height squared (m2) by the weight (kg)”. You have reversed the calculation

BMI=weight (kg) /Height in Meters squared

BMI = weight (kg) / height² (m²) Not the other way round as you stated it.

In general, the methods have some critical issues that need to be fixed, BMI formula incorrect, Fold- change threshold (first location line 229-232, second location (254-255)) is incorrect, kindly rectify that.

RESULTS

Strengths

1. The results were clearly reported, clearly confirmation of matching.

2. P-value is appropriately reported.

3. There is a multi-level taxonomic interpretation.

Weaknesses

1. On line 285, Kindly replace “healthy families” with “healthy controls” because at some points controls were described as health controls, which makes the group description incorrect.

2. On line 314, “192 fecal samples from the HC group (96) and HC patients (96) …

Both groups labeled HC, second should be HD.

CONCLUSION

STRENGTHS

1. The main findings are clearly summarized and aligned with results.

2. On line 439-441, Novelty is reinforced.

3. On line 441-443, The findings are connected to clinical implications

WEAKNESSES

1. ON LINE 436-444, conclusion should provide some takeaways, not to restate discussion-level explanations.

2. Conclusion misses opportunity to highlight methodological strengths already presented in manuscript.

**Reviewer #3:**  The study titled “Integrated Multi-Omics Reveals Dysbiosis in Hemodialysis Patients: A Multi-Center Study” by Zhang et al. addresses an important and timely topic. However, I recommend that the authors make the following edits to improve their manuscript.

1. Page 12, Lines 73-78: The reported number of hemodialysis patients in China (8.44 million) appears implausibly high. Available epidemiological data suggest that the number of patients on dialysis is more likely in the hundreds of thousands rather than millions. The authors should recheck this figure and ensure that the statistic is accurate, or clarify whether this number refers to a broader population (e.g., total CKD prevalence) rather than patients receiving hemodialysis.

2. Page 12, Lines 73-78: The statement that hemodialysis accounts for nearly 90% of patients receiving renal replacement therapy “in most countries” is overly broad not accurate, as the distribution of dialysis modalities varies significantly by region and healthcare system. The authors should revise this statement to be more specific, either by providing region-specific data or qualifying the claim with appropriate references.

3. Pages 12-13, Introduction Section: The introduction would benefit from a stronger rationale for studying gut dysbiosis specifically in hemodialysis patients. The authors should expand this section by emphasizing the clinical relevance of gut microbiome alterations in this population. In particular, prior studies have shown that distinct gut microbial compositions are associated with increased mortality risk among hemodialysis patients and may help identify individuals with a poorer prognosis. Highlighting this prognostic relevance would better justify the importance and clinical implications of the present study.

4. Page 14, Line 122: The matching procedure is unclear. The authors state that 96 hemodialysis patients were paired with 96 family members living with them. It is not clear what is meant by this statement or how the pairing was performed. The authors should clarify what they mean and explain their matching strategy.

5. Page 14, Lines 128-130: The authors state that patients and controls had “identical diet and living conditions,” but it is unclear how this was achieved. Even among cohabitating individuals, dietary variability can exist. The authors should elaborate on how diet and living conditions were assessed and controlled, and clarify the basis for this statement.

6. Page 14, Line 134: The manuscript states that Ethics Committee approval was obtained, but important details are missing. The authors should provide the approval number, specify the type of consent obtained from participants, and indicate whether any waiver of consent was granted, particularly given the retrospective nature of the study.

**Reviewer #4:**  What is novel in your multi-omics integration compared with previous studies?

Does the paired design under identical dietary and living conditions represent the main innovation?

Are these fecal or systemic markers?

How feasible is their use in routine HD practice?

Was diet standardized or monitored?

For how long prior to sampling?

Add a correlation network figure showing microbiome–metabolome interactions.

Include a heatmap of significantly altered metabolites.

Discuss potential interventions:

Probiotics?

Vitamin supplementation?

Dietary modulation?

Clearly define study limitations:

Cross-sectional design; 16S rather than shotgun sequencing; Lack of serum metabolomics; Single-center cohort

**********

what does this mean?). If published, this will include your full peer review and any attached files.

If you choose “no”, your identity will remain anonymous but your review may still be made public.

**Do you want your identity to be public for this peer review?**  For information about this choice, including consent withdrawal, please see our Privacy Policy

Reviewer #1: **Yes:**  Ganesh Praneeth Roy Avula

Reviewer #2: **Yes:**  Shadrack Barffour Awuah

Reviewer #3: No

Reviewer #4: **Yes:**  ahmed alshewered

**********

[NOTE: If reviewer comments were submitted as an attachment file, they will be attached to this email and accessible via the submission site. Please log into your account, locate the manuscript record, and check for the action link "View Attachments". If this link does not appear, there are no attachment files.]

To ensure your figures meet our technical requirements, please review our figure guidelines: https://journals.plos.org/plosone/s/figures

You may also use PLOS’s free figure tool, NAAS, to help you prepare publication quality figures: https://journals.plos.org/plosone/s/figures#loc-tools-for-figure-preparation.

NAAS will assess whether your figures meet our technical requirements by comparing each figure against our figure specifications.

# Author comment: Author response to Decision Letter 1

**DOI:** 10.1371/journal.pone.0355698.r002

10 May 2026

Dear Editor and Reviewers:

A pleasant day to you!

We sincerely thank you for the opportunity to revise our manuscript and for the constructive comments provided by the reviewers. We have carefully addressed all major concerns raised by Reviewers 2, 3, and 4. For initially unclear points, we consulted academic advisors and field professionals to ensure accurate understanding and proper responses. We believe the revised manuscript now meets the standards for publication in PLOS ONE.

We have formatted the revised manuscript according to the PLOS ONE templates, including proper file naming, line numbering, title page, and figure formatting. We have corrected the funding information in the online submission system. The grant number is now consistent: No. ZF2024069. We have regenerated and uploaded high-resolution versions of all requested figures. Clarity has been substantially improved. We have addressed all editorial requirements as follows: no custom code was generated (SPSS/GraphPad Prism used); the funding statement has been amended to include “There was no additional external funding received for this study”; the funders had no role in the study; and all data underlying the findings are fully available within the manuscript and its Supporting Information files. Raw sequencing data were uploaded to a public database to meet transparency requirements. DOI: 10.57760/sciencedb.34128. These updates have been detailed in our revised cover letter.

Below we provide a point-by-point response to each reviewer comment. All changes in the revised manuscript have been highlighted for your convenience.

Thank you once again for your time and valuable input. We look forward to your response.

Sincerely,

Yours,

Hailing Di

Attachment Submitted filename: Response_Letter.docx

# Peer review: Decision Letter 1

**DOI:** 10.1371/journal.pone.0355698.r003

**Authors:** Ze Xiang

26 Jul 2026

Integrated Multi-Omics Reveals Dysbiosis in Hemodialysis Patients: A Multi-Center Study

PONE-D-25-56310R1

Dear Dr. Di,

We’re pleased to inform you that your manuscript has been judged scientifically suitable for publication and will be formally accepted for publication once it meets all outstanding technical requirements.

Within one week, you’ll receive an e-mail detailing the required amendments. When these have been addressed, you’ll receive a formal acceptance letter and your manuscript will be scheduled for publication.

An invoice will be generated when your article is formally accepted. Please note, if your institution has a publishing partnership with PLOS and your article meets the relevant criteria, all or part of your publication costs will be covered. Please make sure your user information is up-to-date by logging into Editorial Manager at Editorial Manager® and clicking the ‘Update My Information' link at the top of the page. For questions related to billing, please contact billing support.

If your institution or institutions have a press office, please notify them about your upcoming paper to help maximize its impact. If they’ll be preparing press materials, please inform our press team as soon as possible -- no later than 48 hours after receiving the formal acceptance. Your manuscript will remain under strict press embargo until 2 pm Eastern Time on the date of publication. For more information, please contact onepress@plos.org.

Kind regards,

Ze Xiang

Academic Editor

PLOS One

Additional Editor Comments (optional):

Reviewers' comments:

Reviewer's Responses to Questions

**Comments to the Author**

Reviewer #3: All comments have been addressed

**********

2. Is the manuscript technically sound, and do the data support the conclusions?

Reviewer #3: Yes

**********

3. Has the statistical analysis been performed appropriately and rigorously?

Reviewer #3: Yes

**********

4. Have the authors made all data underlying the findings in their manuscript fully available?

The PLOS Data policy

Reviewer #3: Yes

**********

5. Is the manuscript presented in an intelligible fashion and written in standard English?

Reviewer #3: Yes

**********

Reviewer #3: (No Response)

**********

what does this mean?). If published, this will include your full peer review and any attached files.

If you choose “no”, your identity will remain anonymous but your review may still be made public.

**Do you want your identity to be public for this peer review?**  For information about this choice, including consent withdrawal, please see our Privacy Policy

Reviewer #3: No

**********

# Editor report: Acceptance letter

**DOI:** 10.1371/journal.pone.0355698.r004

**Authors:** Ze Xiang

PONE-D-25-56310R1

PLOS One

Dear Dr. Di,

I'm pleased to inform you that your manuscript has been deemed suitable for publication in PLOS One. Congratulations! Your manuscript is now being handed over to our production team.

At this stage, our production department will prepare your paper for publication. This includes ensuring the following:

* All references, tables, and figures are properly cited

* All relevant supporting information is included in the manuscript submission,

* There are no issues that prevent the paper from being properly typeset

You will receive further instructions from the production team, including instructions on how to review your proof when it is ready. Please keep in mind that we are working through a large volume of accepted articles, so please give us a few days to review your paper and let you know the next and final steps.

Lastly, if your institution or institutions have a press office, please let them know about your upcoming paper now to help maximize its impact. If they'll be preparing press materials, please inform our press team within the next 48 hours. Your manuscript will remain under strict press embargo until 2 pm Eastern Time on the date of publication. For more information, please contact onepress@plos.org.

You will receive an invoice from PLOS for your publication fee after your manuscript has reached the completed accept phase. If you receive an email requesting payment before acceptance or for any other service, this may be a phishing scheme. Learn how to identify phishing emails and protect your accounts at https://explore.plos.org/phishing.

If we can help with anything else, please email us at customercare@plos.org.

Thank you for submitting your work to PLOS ONE and supporting open access.

Kind regards,

PLOS ONE Editorial Office Staff

on behalf of

Dr. Ze Xiang

Academic Editor

PLOS One
